# Mapping the research landscape of the interactions between obesity and five major complications of diabetes: a bibliometric analysis using knowledge graph visualization

**DOI:** 10.3389/fendo.2025.1626191

**Published:** 2025-10-23

**Authors:** Yutong Liu, Yan Wang, Xiaoming Tian, Tianyu Jiang, Chen Tang, Likun Zhu, Wenze Cui, Wenhuan Song, Chong Ma, Shoujun Song, Mingkun Yu

**Affiliations:** ^1^ Affiliated Traditional Chinese Medicine Hospital of Binzhou Medical College (Binzhou Municipal Traditional Chinese Medicine Hospital), Binzhou, Shandong, China; ^2^ Heilongjiang University of Chinese Medicine, Harbin, Heilongjiang, China; ^3^ Feicheng City People’s Hospital, Feicheng, Shandong, China; ^4^ Binzhou Medical University, Yantai, Shandong, China; ^5^ Affiliated Hospital of Binzhou Medical University, Binzhou, Shandong, China

**Keywords:** obesity, diabetes mellitus, diabetic complications, bibliometric analysis, knowledge graphs, research trends, interaction analysis

## Abstract

**Background:**

Obesity significantly increases the risk of major complications of diabetes, including diabetic kidney disease (DKD), diabetic angiopathy, diabetic peripheral neuropathy, diabetic retinopathy, and diabetic foot ulcers (DFU). Although there is a well-established link between obesity and these complications, a comprehensive bibliometric study is needed to map the research landscape and identify the intellectual structure regarding the interactions between obesity and these complications.

**Purpose:**

This study aimed to systematically map the global research trends, key themes, and emerging frontiers in the interactions between obesity and five major complications of diabetes using bibliometric analysis and knowledge graph visualization.

**Methods:**

A comprehensive bibliometric analysis was conducted via Web of Science, Scopus, and PubMed databases (January 1, 2015, to March 17, 2025) addressing the interplay between obesity and five major complications of diabetes. Using VOSviewer and CiteSpace, the field’s intellectual structure, collaboration networks, and thematic evolution were mapped by analyzing co-citation, keyword co-occurrence, and keyword bursts.

**Results:**

The analysis of 5,475 articles revealed a rapidly growing field, dominated by research on DKD (n=1,571) and diabetic angiopathy (n=1,303), and led by institutions in the USA and China. Thematic network analysis revealed that ‘obesity’, ‘insulin resistance’, and ‘inflammation’ represent the core pathophysiological mechanisms linking all five complications of diabetes. The keyword burst indicated a significant thematic evolution in the field. Specifically, the focus of studies has transitioned from initial studies on foundational associations to more in-depth studies targeting specific molecular pathways (e.g., ‘NF-kappa B’), high-impact therapeutic interventions (‘metabolic surgery’), and distinct patient populations (‘children’). Through co-citation analysis, we found that research on obesity provides a unified intellectual backbone that structurally integrates the disparate research streams of the five major complications of diabetes.

**Conclusion:**

This study quantitatively confirmed that obesity is a scientific nexus for the five major complications of diabetes, shaping a research field characterized by rapid evolution and increasing mechanistic complexity. In conclusion, our findings advocate for a clinical paradigm that establishes weight management as a core component of diabetes treatment, while also guiding future studies toward the systematic clinical translation of mechanism-based interventions.

## 1 Introduction

Globally, obesity and type 2 diabetes mellitus (T2DM) are major public health concerns, affecting millions of lives and leading to premature mortality and substantial socioeconomic burden on healthcare systems worldwide (1, 2). Among modifiable risk factors, obesity not only accelerates the progression of T2DM but also accelerates the development of severe and long-term complications (3).

The development and progression of major complications, specifically diabetic kidney disease (DKD), diabetic angiopathy, diabetic peripheral neuropathy (DPN), diabetic retinopathy (DR), and diabetic foot ulcers (DFU), transform diabetes from a manageable chronic condition into a primary driver of morbidity, disability, and immense healthcare costs (4, 5). Many preclinical and clinical studies attempted to unravel the precise mechanisms by which obesity worsens these complications (6, 7). This process was found to involve a complex network of interacting pathways, yet the central challenge lies in their context-specificity. Specifically, the activation and interplay of these pathways vary significantly based on the affected organ and the specific complication (8). Key nodes in this pathogenic network include insulin resistance, chronic inflammation, oxidative stress, lipotoxicity, and gut dysbiosis (9–11). Therefore, a deeper understanding of their specific functions and complex relationships remains an unmet scientific objective (12). Although previous studies have extensively measured many individual nodes and pathways linking obesity to the specific complications of diabetes, they have largely proceeded in parallel, offering a fragmented body of knowledge (13, 14). Thus, a comprehensive synthesis is necessary to integrate these disparate findings, map the collective intellectual evolution, and chart a unified research agenda across all five complications (15). Despite being insightful, traditional narrative reviews cannot address such a synthetic task, as they lack the quantitative framework needed to systematically map knowledge dynamics, research patterns, and emergent frontiers within a vast and diverse body of scholarship (16).

Bibliometric methods can quantitatively and systematically unveil the architecture, trends, and collaborative frameworks of a scientific discipline (17, 18). This capacity is provided through the systematic analysis of networks formed by authors, institutions, and keywords, offering insights into three key dimensions of the discipline: its historical trajectory, its core intellectual centers, and its prospective research frontiers (19).

Building upon this methodological foundation, the present study applied these bibliometric and visualization techniques to a specific and critical research domain: the intellectual structure and evolution of the interplay between obesity and the five major complications of diabetes, including DKD, diabetic angiopathy, DPN, DR, and DFU, in the literature from 2015 to 2025 (20, 21). Therefore, our study systematically mapped publication trends and global research distribution to identify core scholarly entities and their collaborations, analyze dominant thematic structures and their interconnections, and examine the field’s foundational knowledge base via co-citation analysis. This study provides an objective overview to foster interdisciplinary communication and offers strategies to mitigate this critical public health issue (22–27).

## 2 Methods

### 2.1 Data collection

A systematic search was conducted across three major electronic databases, including the Web of Science Core Collection (WoSCC), Scopus, and PubMed, to identify all relevant studies measuring the association between obesity and the five principal complications of diabetes. We restricted the scope of literature retrieval to publications issued between January 1, 2015, and March 17, 2025. We deliberately selected this 10-year time frame to ensure the inclusion of the contemporary development phase of diabetology, a period that has undergone fundamental transformation due to cardiovascular outcome trials (CVOTs) of novel medications, such as sodium-glucose cotransporter-2 (SGLT2) inhibitors and GLP-1 receptor agonists (GLP1-RAs). By concentrating on this contemporary period, our analysis accurately reflects the current scientific landscape, avoiding dilution from previous studies that predate the paradigm shift initiated by these transformative agents in the management of obesity and complications of diabetes. Thematically, the search targeted studies concerning the five major complications of diabetes, including DPN, DKD, diabetic angiopathy, DR, and DFU. Obesity, as the central focus of this study, was selected based on its well-established role as a key factor with both profound clinical significance and strong pathophysiological links to the complications of diabetes. The employed search strategy integrated general keywords for obesity (e.g., obesity, obese, overweight) with specific medical subject headings (MeSH) for each target complication. The search strategy was designed to combine general obesity-related keywords with specific MeSH terms for each target complication. The full, database-specific search strings are detailed in Appendix 1. All retrieved records were consolidated and standardized using the bibliographic management software EndNote (version X9). A rigorous two-step deduplication protocol was implemented to ensure the uniqueness of the final dataset. First, an automated screening was conducted using the “Find Duplicates” feature in EndNote. Second, a manual review of titles, authors, and publication years was conducted to identify any remaining duplicates. The final consolidated dataset was derived from five independent searches. Publications were deemed eligible for inclusion if they met two primary criteria. Conceptually, they had to address the intersection of obesity and one of the five specific complications, a requirement addressed by using the Boolean operator “AND” to combine the respective term sets. In terms of document type, eligibility was restricted to journal articles, reviews, and conference papers to ensure a comprehensive capture of relevant studies.

### 2.2 Bibliometric indicators

Our bibliometric analysis assessed four distinct dimensions of the research field using corresponding indicators. Research productivity was evaluated based on publication volume (the total number of publications per subfield), and scholarly influence was gauged based on citation count (the cumulative citations received). In addition to tracking publication volume and citations, we determined journal prestige using impact factor (IF) and identified emerging research frontiers through keyword burst detection, which pinpoints terms with a sudden surge in usage. Furthermore, we incorporated the h-index, g-index, and m-index for a more nuanced assessment of scholarly impact. Respectively, these metrics quantify the balance of productivity and citation impact, assign greater weight to highly-cited works, and normalize impact by the length of academic career.

### 2.3 Burst detection

Using CiteSpace, we conducted keyword burst detection to identify research trends and new topics by computing each keyword’s “burst strength” and mapping its active period from 2015 to March 17, 2025. This analysis quantifies the evolving research focus on the pivotal pathophysiological mechanisms linking obesity to the complications of diabetes by identifying keywords with a rapid surge in frequency. These burst keywords function as empirical signposts, quantitatively mapping the trajectory of scientific findings regarding the mechanistic links between obesity and the complications of diabetes.

### 2.4 Visualization of collaboration networks

We employed network visualization as a primary analytical tool to comprehensively map the research landscape. Specifically, we generated collaboration networks to reveal social structures, produced keyword co-occurrence networks to identify thematic clusters, and provided co-citation networks to uncover the underlying intellectual foundations of the field. Co-citation networks were generated to identify foundational intellectual clusters by linking items (primarily documents or authors) frequently cited together in other publications. Similarly, keyword co-occurrence networks were generated to map prevalent research themes by connecting keywords that frequently appear in conjunction. Collaboration networks were also constructed to visualize the social dimension of the research landscape, detailing the relationships between contributing authors, institutions, and countries. We also used visualization networks to identify key research clusters, leading authors, and major collaborative entities and conducted a journal co-citation analysis to ascertain the most influential scholarly journals shaping this field of research. We analyzed the distribution of publications across different journals. The academic influence of these journals was evaluated based on their IF and JCR classification. All journal metrics were obtained from the Journal Citation Reports (JCR) 2023, released by Clarivate.

### 2.5 Data analysis tools

For data processing, network construction, and visualization, we employed two widely recognized bibliometric software packages, VOSviewer (version 1.6.x) and CiteSpace (version 6.x.x), which can help specifically analyze large bibliographic datasets, construct complex networks (e.g., co-authorship, co-citation, and keyword co-occurrence), identify thematic clusters, and visualize temporal trends in a research landscape. All analyses were conducted with specific parameters to ensure reproducibility. In VOSviewer, networks were constructed based on the full counting method, with minimum thresholds set at 5 documents for countries/organizations and 10 occurrences for keywords. Clusters were identified using their native modularity-based algorithm. In CiteSpace, temporal analyses were conducted utilizing 1-year time slices, and we selected the top 50 items per slice (Top N = 50) as network nodes, followed by Pathfinder network pruning. Keyword bursts were detected using Kleinberg’s algorithm with a minimum duration of 2 years.

## 3 Results

### 3.1 Literature search and publication trends

After removing duplicate entries, 5,475 unique articles were included in the analysis, distributed across DKD (n=1,571), diabetic angiopathy (n=1,303), DPN (n=953), DR (n=870), and DFU (n=778). The detailed literature screening process is illustrated in the PRISMA flow diagram (**Supplementary Figure S1**).

**Figure 1:**
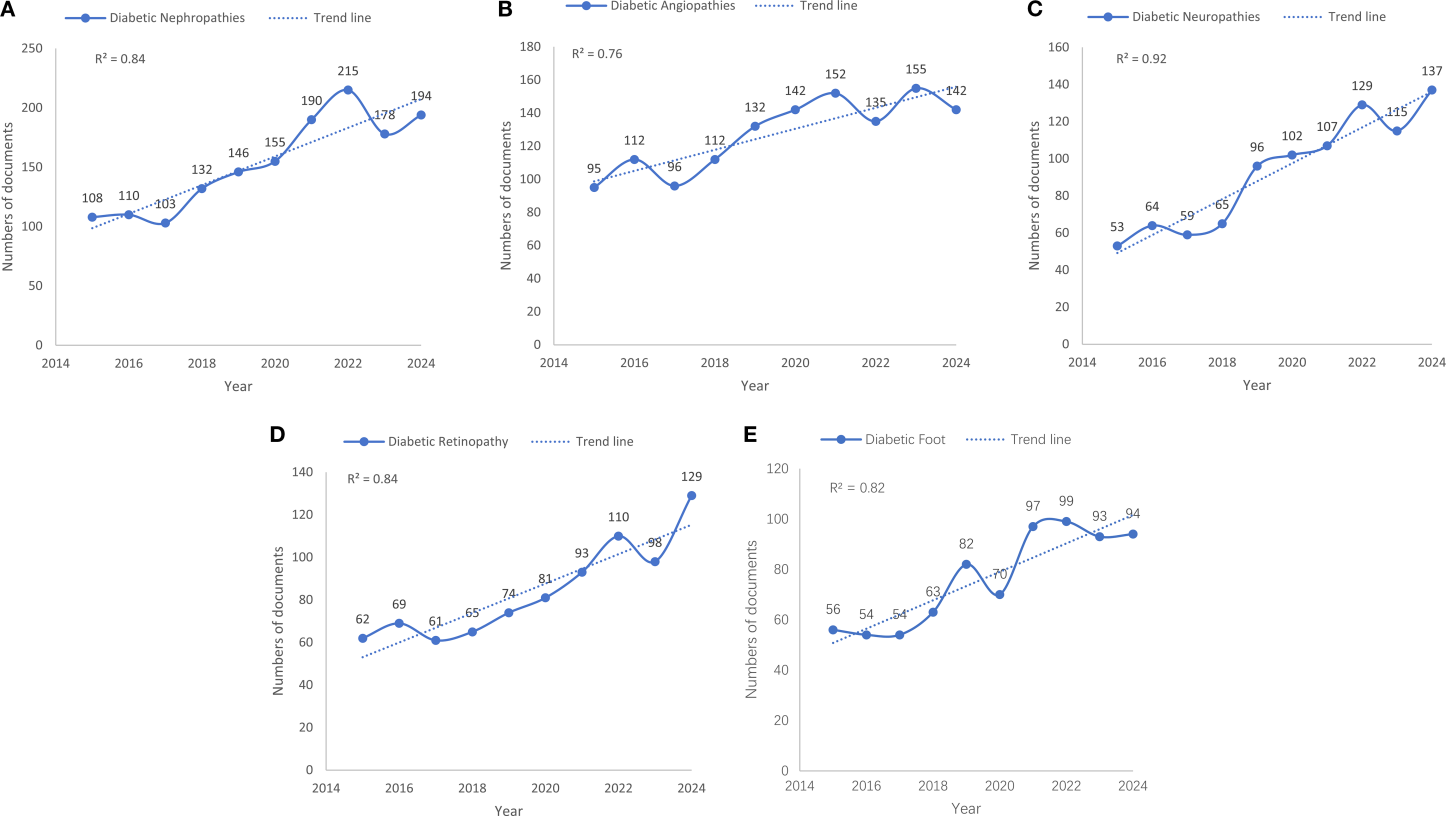
Publication trends of five major complications of diabetes and obesity (2015–2024): **(A)** DKD, **(B)** Diabetic angiopathy, **(C)** DPN, **(D)** DR, and **(E)** DFU.

**Figures 1A–E** present the annual publication trends for the five complications from 2015 to 2024. Although our search was extended to March 2025, the trend analysis and reported R² values were intentionally based on complete annual data from 2015 to 2024. This methodological choice ensured statistical integrity by preventing partial 2025 data from skewing the regression analysis.

Overall, all five fields exhibited a positive growth trend. Key comparative findings were as follows:

DKD (**Figure 1A**): Research on DKD consistently held the highest publication volume, showing a strong and steady increase (R² = 0.84), peaking in 2022 with 215 articles.

Diabetic angiopathy (**Figure 1B**): This field ranked second in terms of volume and displayed a positive but more variable growth trend (R² = 0.76), evidenced by significant fluctuations around the trend line.

DPN (**Figure 1C**): While having a lower publication volume, DPN exhibited the most consistent and steepest growth trajectory, confirmed by the highest coefficient of determination (R² = 0.92), with a clear acceleration after 2018.

DR (**Figure 1D**): Research on DR also showed a robust upward trend (R² = 0.84), with a notable acceleration from 2019.

DFU (**Figure 1E**): Research on DFU displayed a positive but fluctuating growth pattern (R² = 0.82), characterized by a sharp increase after 2018 and a peak in 2022.

### 3.2 Geographic and institutional distribution of research

Geographic and institutional analysis (**Table 1**) revealed that the United States and China were the most prolific contributors in these areas. The US led in terms of the number of publications related to DFU (n=245), DPN (n=268) and diabetic angiopathy (n=310), while China was at the forefront of research in the field of DKD (n=381) and DR (n=201). India, the UK, Germany, Japan, and Australia also frequently appeared among the top ten countries. High publication output from these countries correlated with high citation counts, suggesting a significant research impact. Notably, institutions such as Harvard Medical School (USA), the University of Michigan (USA), and Shanghai Jiao Tong University (China) ranked among the top institutions, reinforcing the leadership positions of their countries.

**Table 1:** Top 10 countries/ regions and organizations related to DKD, diabetic angiopathy, DPN, DR, and DFU.

Type	Rank	Country/region	Avg. pub. year	Documents	Citations	Rank	Organization	Avg. pub. year	Documents	Citations
DKD	1	People's Republic of China	2021.12	381	6384	1	Shanghai Jiao Tong Univ	2021.80	25	281
	2	USA	2019.52	298	8230	2	Monash Univ	2020.23	22	515
	3	Japan	2019.60	121	2340	3	Univ Michigan	2020.62	21	686
	4	India	2021.61	93	1571	4	Huazhong Univ Sci & Technol	2022.30	20	233
	5	United Kingdom	2020.00	72	2307	5	Sun Yat Sen Univ	2020.90	19	295
	6	Spain	2019.80	71	2312	6	Southern Med Univ	2022.00	16	135
	7	South Korea	2020.21	68	1171	7	Korea Univ	2020.47	15	183
	8	Australia	2019.59	66	1759	8	Univ Washington	2020.00	15	647
	9	Italy	2020.28	64	2055	9	China Med Univ	2019.29	14	205
	10	Germany	2019.39	61	1901	10	Univ Coll Dublin	2020.00	14	190
Diabetic angiopathy	1	USA	2019.42	310	12104	1	Harvard Med Sch	2020.72	25	1672
	2	People's Republic of China	2020.58	223	4943	2	Shanghai Jiao Tong Univ	2022.24	17	223
	3	United Kingdom	2019.91	101	3030	3	Heidelberg Univ	2019.27	15	741
	4	India	2021.44	90	1805	4	Univ Coll Dublin	2018.27	15	652
	5	Germany	2019.17	66	3458	5	Univ Copenhagen	2019.20	15	2178
	6	Italy	2019.35	57	2875	6	Chinese Univ Hong Kong	2020.57	14	650
	7	Japan	2019.29	52	1306	7	Univ Manchester	2021.93	14	282
	8	Australia	2019.60	45	1583	8	Univ Missouri	2018.71	14	486
	9	Brazil	2019.22	45	937	9	Imperial Coll London	2018.62	13	549
	10	Spain	2020.36	45	1008	10	Maastricht Univ	2020.15	13	747
DPN	1	USA	2020.31	268	9456	1	Univ Michigan	2020.29	52	3290
	2	People's Republic of China	2021.56	138	2484	2	Univ Manchester	2020.72	25	422
	3	United Kingdom	2020.09	79	2839	3	Univ Iowa	2017.56	16	591
	4	India	2021.55	73	912	4	Weill Cornell Med Qatar	2020.93	15	288
	5	Germany	2019.38	56	2001	5	German Ctr Diabet Res Dzd	2021.67	12	222
	6	Japan	2019.77	34	581	6	Heinrich Heine Univ Dusseldorf	2021.67	12	530
	7	Italy	2020.63	32	2314	7	Aarhus Univ	2020.64	11	356
	8	Australia	2020.77	30	547	8	Aarhus Univ Hosp	2020.27	11	515
	9	France	2019.52	25	959	9	Mayo Clin	2020.73	11	313
	10	Brazil	2019.67	24	364	10	Harvard Med Sch	2020.78	9	1193
DR	1	People's Republic of China	2021.14	201	3883	1	Sun Yat Sen Univ	2021.26	23	346
	2	USA	2020.23	151	5755	2	Shanghai Jiao Tong Univ	2021.86	21	376
	3	India	2021.01	71	960	3	Heidelberg Univ	2019.82	11	312
	4	United Kingdom	2020.10	51	1167	4	Natl Univ Singapore	2018.18	11	375
	5	Australia	2019.96	44	1095	5	Sungkyunkwan Univ	2019.50	10	112
	6	Japan	2020.25	40	609	6	Univ Melbourne	2020.80	10	219
	7	Saudi Arabia	2020.50	34	380	7	Univ Sydney	2018.10	10	408
	8	South Korea	2019.82	33	794	8	Catholic Univ Korea	2018.67	9	112
	9	Germany	2019.25	32	1327	9	Univ Alabama Birmingham	2020.44	9	695
	10	Iran	2021.33	30	370	10	Chinese Univ Hong Kong	2020.63	8	306
DFU	1	USA	2020.02	245	6789	1	Harvard Med Sch	2020.00	13	317
	2	People's Republic of China	2021.01	89	2136	2	Univ Michigan	2019.90	10	463
	3	India	2020.85	58	1125	3	Univ Illinois	2020.22	9	266
	4	United Kingdom	2019.20	50	1516	4	Univ Toronto	2017.88	8	160
	5	Germany	2019.29	38	685	5	Monash Univ	2020.00	7	150
	6	Australia	2019.89	36	820	6	Northwestern Univ	2018.29	7	979
	7	Italy	2020.19	31	796	7	Shanghai Jiao Tong Univ	2019.14	7	563
	8	Saudi Arabia	2020.96	22	362	8	Boston Univ	2018.83	6	161
	9	Spain	2019.55	22	627	9	Univ Washington	2017.00	6	92
	10	Brazil	2020.05	21	461	10	Huazhong Univ Sci & Technol	2021.60	5	56

### 3.3 Author collaboration networks

**Figure 2:**
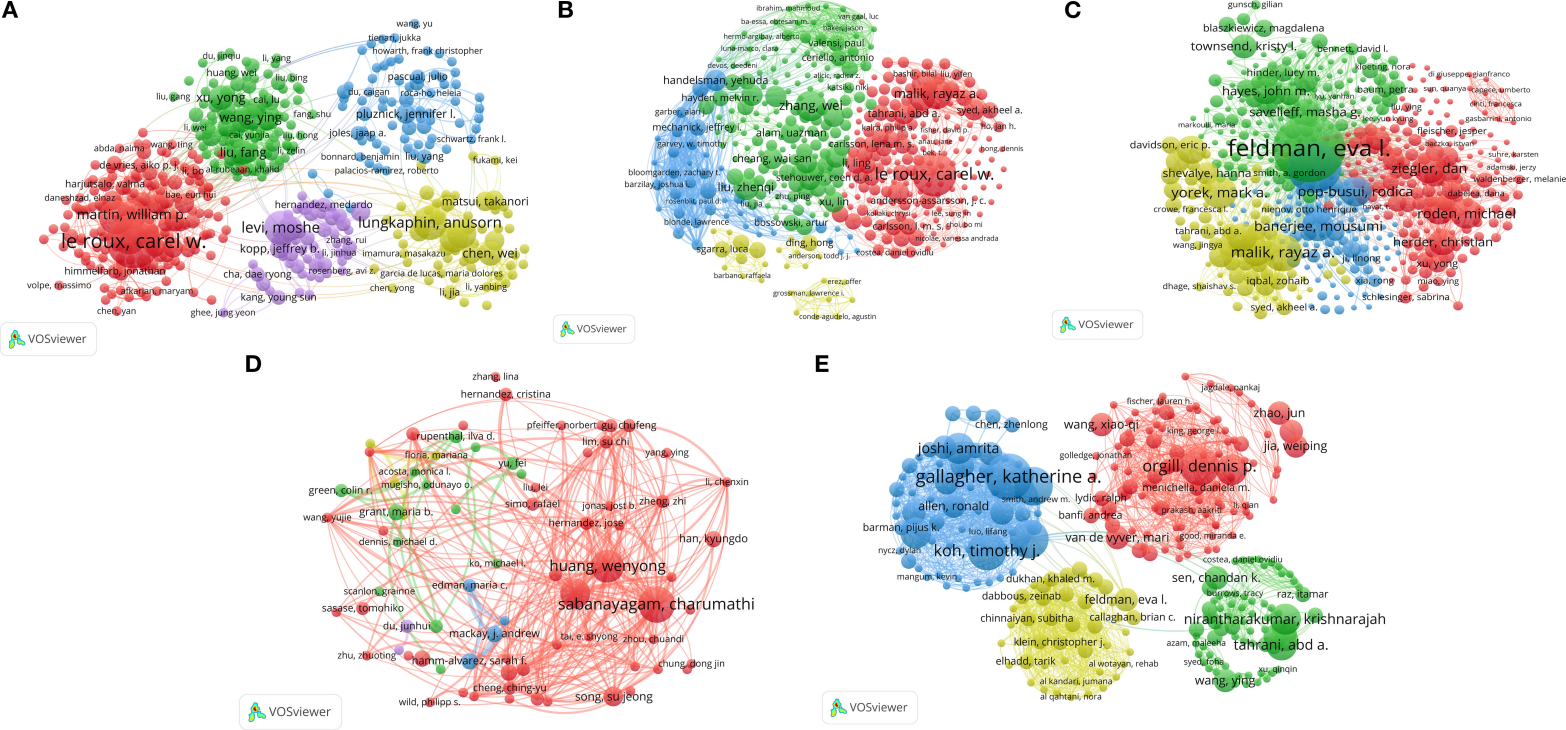
Author coupling network of studies on five types of diabetic complications and obesity: **(A)** DKD, **(B)** Diabetic angiopathy, **(C)** DPN, **(D)** DR, and **(E)** DFU.

**Figure 2A** (DKD) reveals several highly interconnected research clusters. A prominent red network was found around authors such as Le Roux CW and Martin P, who focused on obesity interventions, particularly metabolic surgery and its effect on DKD. Furthermore, a large green network, associated with researchers such as Wang Y and Liu F, represents a research community concentrating on different aspects of DKD, like its epidemiology and renal mechanisms, suggesting a probable geographical focus in China.

**Figure 2B** displays the collaboration network for diabetic angiopathy, revealing a complex structure characterized by a prominent large green cluster. This cluster comprised prominent researchers, including Zhang W, Cheang WS, and Ceriello A, who focused on foundational pathophysiological mechanisms, such as oxidative stress and endothelial function. The green network was strongly interconnected with the red network. The latter features prominent authors, including Le Roux CW. This connection highlights the strong association and potential interplay between research focused on fundamental mechanisms of diabetic angiopathy and studies concerning obesity and metabolic surgery. Moreover, the presence of a distinct blue cluster, comprising Handelsman Y, suggests the focus of the research endeavor on clinical guideline development and the formulation of comprehensive management strategies.

**Figure 2C** illustrates the collaboration network for DPN, which displays a structure significantly more concentrated than those observed for other complications. In this structure, a dense green cluster was organized around Feldman EL, highlighting her central role in fundamental research on DPN. In contrast, a red network, featuring researchers such as Ziegler D and Pop-Busui R, represented leading groups focusing on the clinical and epidemiological aspects of DPN. The strong inter-cluster connectivity highlights the close interactions between basic and clinical research in this subfield, emphasizing the integration of foundational and applied scientific efforts.

**Figure 2D** (DR) reveals a multi-centric research landscape. A prominent red cluster, featuring researchers such as Sabanayagam C, Huang W, and Song SJ, underscores their leadership in ophthalmic epidemiology, with a focus on large cohort studies examining the prevalence of DR and its risk factors, including obesity. Additionally, other clusters, such as the green one led by Grant MB, explored various aspects, including the effect of interventions on the vascular mechanisms involved in DR, highlighting the diverse research efforts in this field.

**Figure 2E** (DFU) illustrates prominent, interconnected blue and red networks, led by Gallagher KA, Koh TJ, and Orgill DP. These networks indicated a core focus on diabetic wound healing, inflammation, and related mechanisms. Additional clusters, such as the yellow cluster led by Feldman EL and the green cluster under Nirantharakumar K, suggested associations with neuropathy and connections to epidemiological approaches utilizing large datasets. This structure highlights the multidisciplinary nature of DFU research, integrating clinical, mechanistic, and epidemiological perspectives.

### 3.4 Analysis of intellectual foundations: co-cited authors

**Figure 3:**
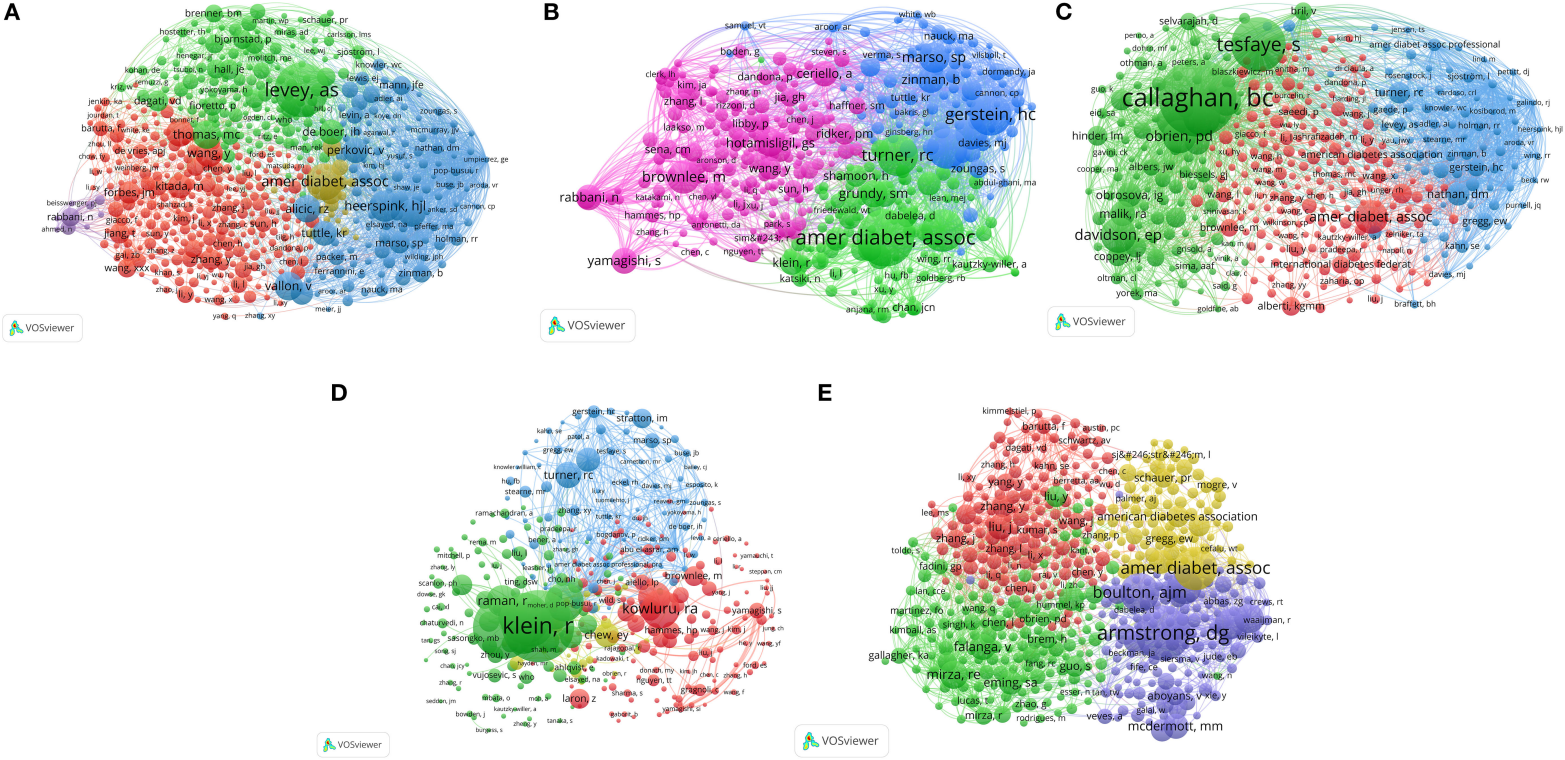
Author co-citation network of studies on five types of diabetic complications and obesity: **(A)** DKD, **(B)** Diabetic angiopathy, **(C)** DPN, **(D)** DR, and **(E)** DFU.

Co-cited author analysis (**Figure 3**, **Table 2**) identified researchers who were frequently co-cited, revealing the intellectual foundations and key figures in each subfield. This analysis identified influential authors and seminal works pivotal to shaping the research landscape in this area. Furthermore, it illuminated the interconnectedness of concepts and the evolution of knowledge across various domains linking obesity and complications of diabetes.

**Table 2:** Top 10 authors and co-cited authors related to DKD, diabetic angiopathy, DPN, DR, and DFU.

Type	Rank	Author	Avg. pub. year	H_index	G_index	M_index	Documents	Citations	Rank	Co-cited author	Citations
DKD	1	Le Roux, CW	2020.00	10	16	1.00	11	163	1	Levey, AS	230
	2	Lee, EY	2019.50	9	14	0.82	8	130	2	ADA	141
	3	Bjornstad, P	2020.00	9	14	0.82	7	305	3	Heerspink, HJL	116
	4	Chung, CH	2019.43	7	12	0.70	7	118	4	Vallon, V	103
	5	Docherty, NG	2019.71	8	10	0.73	7	82	5	Thomas, MC	102
	6	Groop, PH	2021.86	8	8	0.80	7	99	6	Gerstein, HC	100
	7	Koya, D	2018.43	7	8	0.64	7	200	7	Wang, Y	100
	8	Levi, M	2017.14	6	8	0.55	7	342	8	Sharma, K	98
	9	Lim, SC	2018.43	7	7	0.88	7	136	9	Alicic, RZ	97
	10	Lungkaphin, A	2020.14	5	7	0.45	7	192	10	Perkovic, V	89
Diabetic angiopathy	1	Le Roux, CW	2017.67	10	12	0.91	9	267	1	ADA	149
	2	Feldman, EL	2020.14	5	5	0.56	7	338	2	Sjöström, L	106
	3	Malik, RA	2021.33	5	5	0.56	6	131	3	Nathan, DM	105
	4	Adam, S	2021.80	5	5	0.56	5	123	4	Gerstein, HC	104
	5	Liu, Z	2020.80	4	4	0.44	5	81	5	Turner, RC	101
	6	Soran, H	2021.80	4	4	0.44	5	123	6	Defronzo, RA	99
	7	Yorek, MA	2017.80	4	4	0.44	5	110	7	Schauer, PR	95
	8	Alam, U	2022.50	2	3	0.18	4	33	8	Brownlee, M	78
	9	Aroor, AR	2018.00	2	2	0.18	4	98	9	Holman, RR	75
	10	Cheang, WS	2022.00	2	2	0.18	4	29	10	Marso, SP	71
DPN	1	Feldman, EL	2020.03	25	36	2.27	36	1779	1	Callaghan, BC	373
	2	Callaghan, BC	2020.38	17	24	1.70	24	1196	2	Ziegler, D	222
	3	Malik, RA	2020.93	13	18	1.30	15	398	3	Tesfaye, S	221
	4	Yorek, MA	2017.25	11	12	1.00	12	482	4	Pop-Busui, R	209
	5	Banerjee, M	2019.82	9	12	0.90	11	561	5	Smith, AG	148
	6	Pop-Busui, R	2020.00	8	11	0.80	11	627	6	Dyck, PJ	136
	7	Roden, M	2021.36	9	10	0.82	11	185	7	Feldman, EL	126
	8	Ziegler, D	2020.18	9	10	0.82	11	217	8	Vinik, AI	118
	9	Boenhof, GJ	2021.10	8	10	0.89	10	165	9	Davidson, EP	117
	10	Obrosov, A	2017.40	8	9	0.73	10	437	10	ADA	107
DR	1	Sabanayagam, C	2018.46	9	11	0.82	11	432	1	Sabanayagam, C	432
	2	Huang, W	2023.00	8	10	0.73	10	92	2	Wong, TY	297
	3	Wong, TY	2018.78	5	9	1.25	9	297	3	Wang, JJ	213
	4	Song, SJ	2019.17	5	6	0.63	6	62	4	Lim, SC	150
	5	Grant, MB	2021.40	4	5	0.36	5	130	5	Lamoureux, EL	149
	6	Hammes, HP	2018.20	4	4	0.44	5	187	6	Cheng, CY	137
	7	Han, K	2018.80	4	4	0.36	5	57	7	Kumari, N	122
	8	Wang, W	2022.60	3	4	0.27	5	113	8	Li, LJ	120
	9	Cheng, CY	2018.00	3	4	0.27	4	137	9	Wong, TY	116
	10	Du, J	2016.50	2	4	0.18	4	130	10	Man, REK	105
DFU	1	Gallagher, KA	2019.33	6	6	0.55	6	381	1	Armstrong, DG	83
	2	Davis, FM	2020.20	6	6	0.55	5	226	2	ADA	59
	3	Koh, TJ	2021.60	5	5	0.56	5	151	3	Boulton, AJM	51
	4	Kunkel, SL	2019.80	3	5	0.43	5	308	4	Tesfaye, S	50
	5	Joshi, A	2017.50	3	3	0.43	4	243	5	Lipsky, BA	42
	6	Wolf, SJ	2021.00	3	3	0.27	4	153	6	Dyck, PJ	36
	7	Allen, R	2017.33	3	3	0.27	3	262	7	Lavery, LA	35
	8	Bermick, J	2017.67	3	3	0.27	3	107	8	Sen, CK	35
	9	Feldman, EL	2017.67	3	3	0.27	3	230	9	Bus, SA	34
	10	Gudjonsson, JE	2021.33	3	3	0.27	3	117	10	Ziegler, D	34

The analysis of DKD in **Figure 3A**, **Table 2** indicated a mature research field characterized by a robust foundational knowledge basis. In this context, the green cluster prominently featured the seminal contributions of Levey AS regarding renal function assessment, signifying the foundational status of such works in the field. The yellow cluster, associated with the American Diabetes Association (ADA), corresponded to the research area of clinical practice guidelines with a specific focus on weight management. Additionally, the blue cluster, led by Heerspink HJL, pertained to research on novel treatments (e.g., SGLT2 inhibitors). Specifically, these studies investigated the relationship between the weight-lowering effects and nephroprotective properties of these agents. Furthermore, Le Roux CW was identified as an influential author in this network, since he contributed 11 publications that garnered 163 citations.

As illustrated in **Figure 3B** and detailed in **Table 2**, the network structure for diabetic angiopathy exhibited the significant impact of large CVOTs on the research landscape in the field. The blue cluster, including researchers such as Gerstein HC, Marso SP, and Zinman B, represents studies focused on the development and clinical application of novel hypoglycemic agents, particularly GLP-1RAs and SGLT2 inhibitors. Linked with the ADA, Turner RC (UKPDS), and Grundy SM (metabolic syndrome), the green cluster signified the critical importance of epidemiology and guideline-driven risk management strategies in this field. Concurrently, basic research, represented by the red and pink clusters (featuring Hotamisligil GS, Brownlee M, and Libby P), provided key mechanistic insights into the pathogenesis of diabetic angiopathy. Additionally, Le Roux CW, with 9 publications and 267 citations, was recognized for his significant influence in this field.

The analysis of DPN revealed a concentrated network structure, indicating effective knowledge integration facilitated by key experts in the field (**Figure 3C**, **Table 2**). The green cluster, including Tesfaye S and Callaghan BC, focused on the pathogenesis and diagnosis of DPN. Meanwhile, the blue cluster, featuring Nathan DM, Gerstein HC, and Wing RR, provided evidence from major trials on glycemic control and lifestyle interventions. The red cluster, including Brownlee M, Alberti KGMM, and the ADA/IDF, addressed basic mechanisms, definitions, and guidelines. The significant influence of Feldman EL, evidenced by her 36 publications and 1779 citations, underscored the importance of metabolic factors, including obesity, in DPN research.

The analysis of DR (**Figure 3D**, **Table 2**) revealed clusters driven by specific methodologies, inconsistent with the structure observed for DPN. The red cluster, featuring co-cited authors, such as Kowluru RA, Brownlee M, and Yamagishi SI, focused on pathophysiological aspects, like oxidative stress and inflammation. In contrast, the large green cluster, led by Klein R and Raman R, suggests epidemiological studies and imaging-based diagnostics. The blue cluster, including prominent trialists, like Turner RC, and Gerstein HC, focused on major clinical trials and the development of guidelines for the comprehensive management of diabetes. Sabanayagam C, with 11 publications and 432 citations, was recognized as an influential figure in this field (**Table 2**).

The analysis of DFU (**Figure 3E**, **Table 2**) reflected a strong orientation toward clinical practice and problem-driven multidisciplinary integration. The network was composed of distinct clusters, each representing a specific aspect of DFU research. The red cluster, featuring researchers such as Liu Y and Kahn SE, focused on basic pathophysiological mechanisms. The green cluster, led by Falanga V and Brem H, was dedicated to wound healing biology and treatment. The blue cluster, including Armstrong DG and Boulton AJM, emphasized clinical diagnosis, management, and prevention strategies. Additionally, the yellow cluster, involving ADA, Gregg EW, and Schauer PR, integrated guidelines, epidemiology, and macro-interventions. Notably, Gallagher KA, with six publications and 381 citations, was recognized as an influential figure in the field due to her research on inflammation and healing.

### 3.5 Analysis of intellectual foundations: core journals and influential references

**Figure 4:**
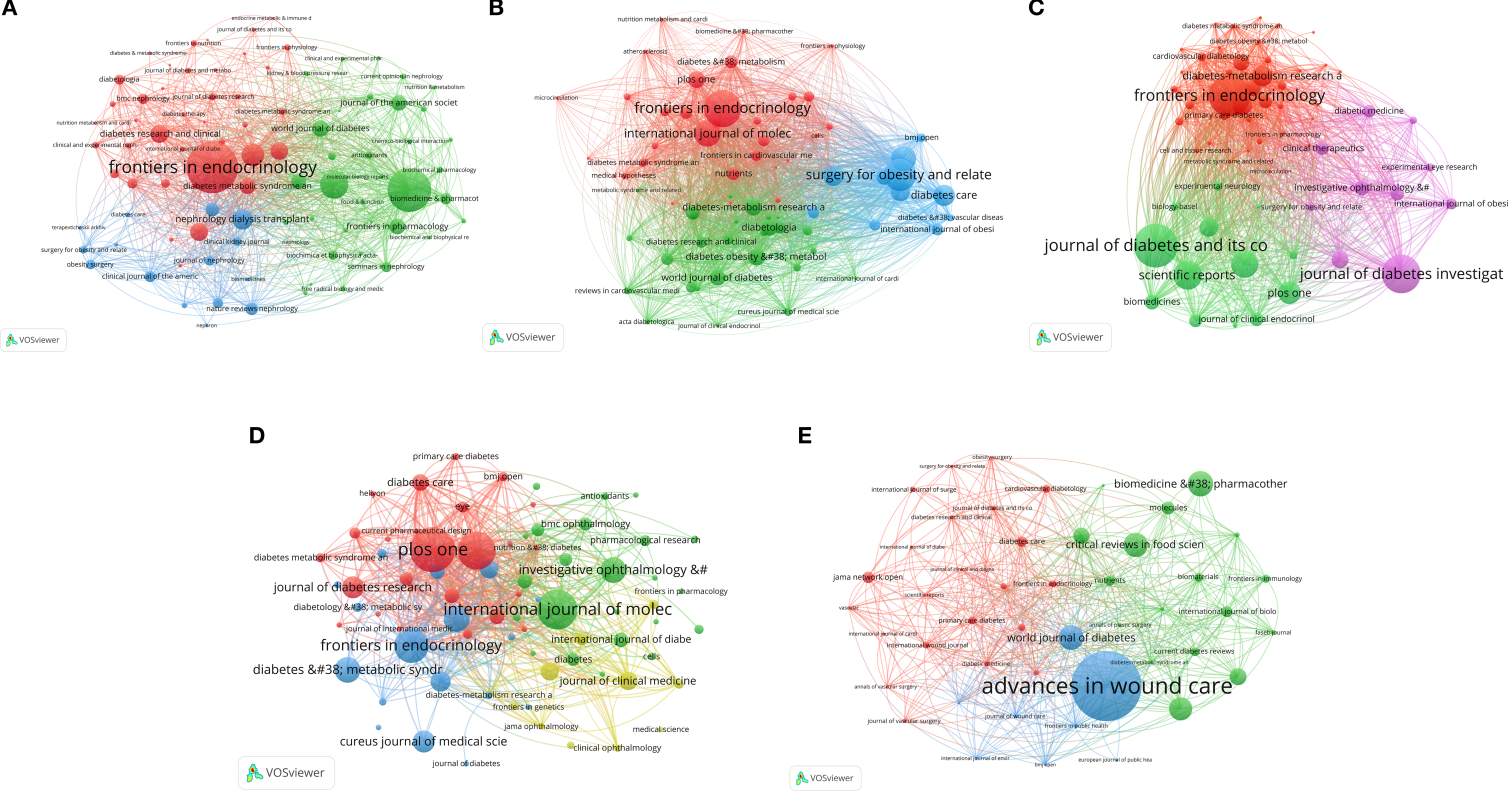
Journal coupling network of studies on five types of diabetic complications and obesity: **(A)** DKD, **(B)** Diabetic angiopathy, **(C)** DPN, **(D)** DR, and **(E)** DFU.

The analysis of journal citation patterns (**Figure 4**, **Supplementary Table S1**) revealed the key publication venues and thematic focuses in the field of obesity and diabetic complications. This analysis identified the most frequently cited journals, reflecting their significant impact and relevance in shaping the discourse and advancing knowledge in these research domains. By identifying these venues, researchers can better understand the dissemination of findings and the development of thematic trends over time.

Regarding DKD, “Frontiers in Endocrinology” emerged as a key journal, effectively linking endocrine perspectives with nephrology-focused publications, such as the “Journal of the American Society of Nephrology” (**Figure 4A**, **Supplementary Table S1**). The “International Journal of Molecular Sciences” with 37 publications and the highest citation count, and “PLoS One” with 39 publications and the highest volume, underscore the significance of research on molecular mechanisms and clinical practice. Additionally, “Frontiers in Endocrinology” with 37 publications, was noted for its high productivity in this field.

Regarding diabetic angiopathy, “Frontiers in Endocrinology” exhibited high centrality, effectively linking basic endocrine and metabolic research (red cluster) to clinical diabetology (green cluster) and interventional studies (blue cluster) (**Figure 4B**, **Supplementary Table S1**). In particular, journals such as “Surgery for Obesity and Related Diseases” and “Diabetes Care” focused on interventional studies. The “International Journal of Molecular Sciences” was significantly influential at the molecular level. “Frontiers in Endocrinology” (29 publications, 541 citations, IF: 3.9, Q1) and “International Journal of Molecular Sciences” (27 publications, 534 citations, IF: 4.9, Q1) led in terms of both output and impact, indicating a strong focus on the endocrine and molecular mechanisms underlying vascular complications and their clinical translation.

Regarding DPN, “Frontiers in Endocrinology” served as a central hub, effectively linking basic endocrine and metabolic research (red cluster) to clinical studies, which were particularly published by the “Journal of Diabetes Complications” (green cluster), and treatment exploration, particularly published by the “Journal of Diabetes Investigation” (purple cluster) (**Figure 4C**, **Supplementary Table S1**). With 26 publications and 267 citations, “Frontiers in Endocrinology” led in terms of both output and influence, underscoring the need for translating endocrine and metabolomic approaches into clinical strategies.

Regarding DR, “PLoS One” serves as a central hub due to its broad scope, effectively linking basic research from journals, such as “International Journal of Molecular Sciences” and “Frontiers in Endocrinology”, to studies related to clinical diabetology published by “Diabetes Care” and “Diabetologia” (**Figure 4D**, **Supplementary Table S1**). “PLoS One” led in terms of publication volume with 25 articles and 588 citations, highlighting its significant impact in the field. “International Journal of Molecular Sciences”, with the second-highest volume (22 publications), underscored pathophysiological molecular mechanisms. It primarily focused on understanding how obesity-related metabolic dysregulation drives DR through molecular pathways.

A distinct pattern was observed regarding DFU (**Figure 4E**, **Supplementary Table S1**). “Advances in Wound Care” (blue cluster) played a central role in the science and practice of wound healing, while “Diabetes Care” (red cluster) served as the authoritative link to clinical diabetology. These journals dominated in terms of citations and impact, with “Advances in Wound Care” receiving 1,367 citations (IF 5.8, Q1) and “Diabetes Care” receiving 1,129 citations (IF 14.8, Q1), establishing them as key knowledge sources. This indicates a research frontier that combines advanced wound care with a systematic analysis of DFU pathogenesis in the context of obesity from both clinical and endocrine perspectives. “Journal of Foot & Ankle Surgery” led in terms of publication number with 16 articles, but it had a lower IF (1.3, Q4), suggesting that it prioritized volume over impact.

**Figure 5:**
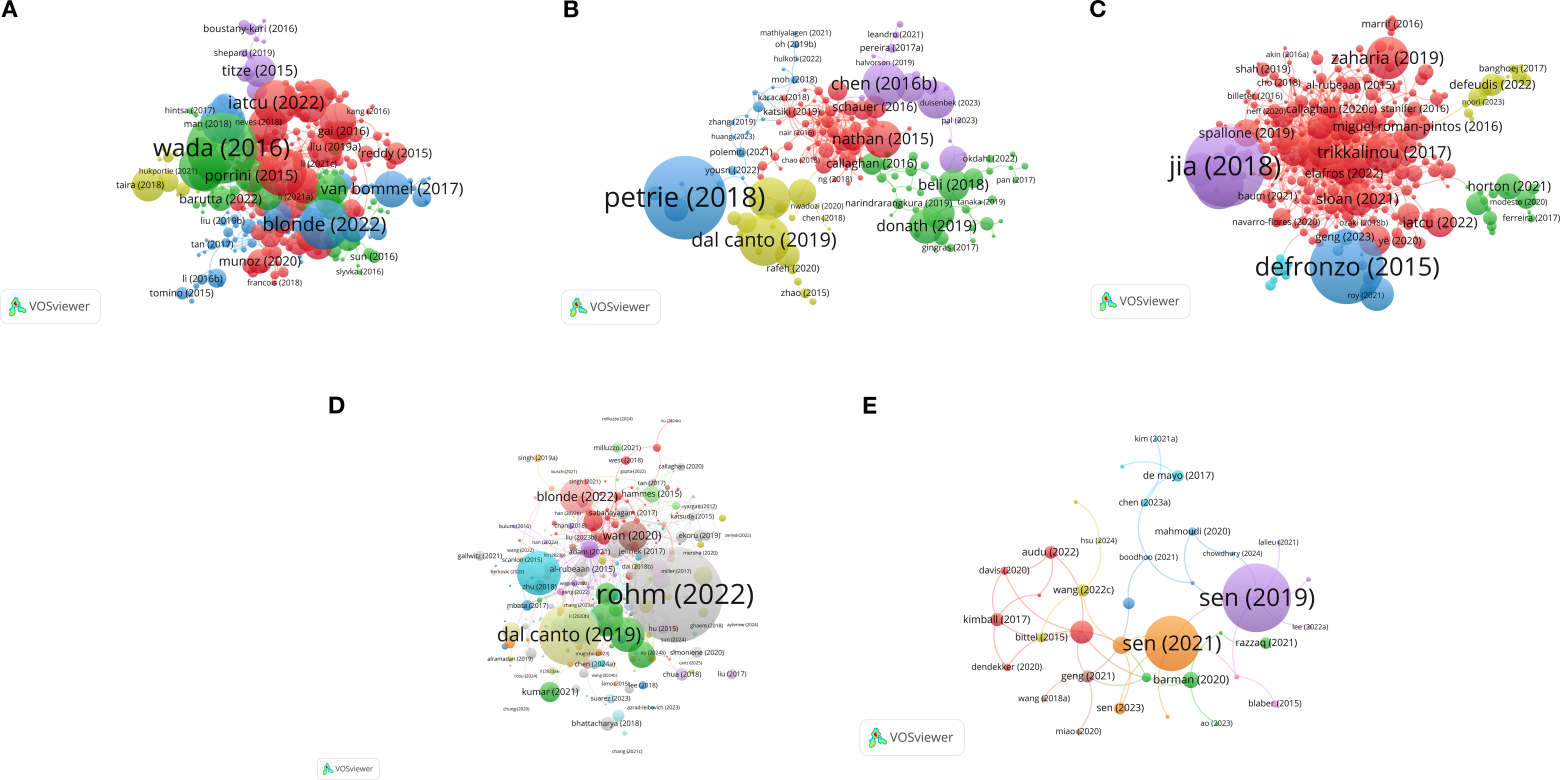
Citation network for studies linking obesity to five major complications of diabetes: **(A)** DKD, **(B)** Diabetic angiopathy, **(C)** DPN, **(D)** DR, and **(E)** DFU.

The analysis of highly cited individual references (**Supplementary Table S2**) (28–75), combined with conceptual network visualizations (**Figure 5**), revealed specific foundational contributions and research focuses in the field.

Foundational knowledge in DKD research was provided by several key works, including the assessment of renal function by Levey AS et al. (28), Ann Intern Med), which garnered 101 citations and pathophysiological reviews by Alicic RZ et al. (29), Clin J Am Soc Nephrol), receiving 75 citations. Additionally, descriptions of obesity-related glomerulopathy by Dagati VD et al. (30), Nat Rev Nephrol) received 61 citations. Trials offering therapeutic breakthroughs, such as the CREDENCE trial by Perkovic V et al. (31), N Engl J Med), also contributed significantly with 61 citations. The work of influential authors who were frequently co-cited represents significant extensions and elaborations upon these foundational studies (**Figure 5A**).

Landmark trials provided a basis for research on diabetic angiopathy, having both established key principles of glycemic control and demonstrated the cardiovascular benefits of novel therapeutic agents. The UKPDS 33 trial, published by Turner RC et al. in The Lancet (1998), garnered 53 citations (within the studied network/period) primarily due to its pivotal role in establishing foundational strategies for glycemic control. Likewise, the LEADER trial (Marso SP et al. (32), N Engl J Med) with 50 citations highlighted the cardiovascular advantages of novel therapeutic agents. **Figure 5B** highlights influential co-cited authors whose research advanced the field by exploring two key areas: the intricate vascular pathology related to obesity and the assessment of innovative therapeutic approaches, such as metabolic surgery.

Authoritative guidelines and position statements significantly affected the direction and priorities of the DPN research landscape. Due to its importance, the ADA position statement on DPN (Pop-Busui R et al. (33), Diabetes Care) received 103 citations. The consensus report by Tesfaye S et al. (34), Diabetes Care) on DPN definition and diagnosis was cited 78 times for providing a key foundational framework. The studies conducted by influential co-cited authors (**Figure 5C**), which primarily delved into the pathogenesis and risk factors of DPN, effectively expanded the established foundational framework.

Regarding DR, the global epidemiological review by Yau JWY et al. (35), Diabetes Care) received 97 citations, and the clinical overview by Cheung N et al. (36), Lancet) was cited 73 times for its comprehensive summary. Other notable foundational works included the international grading standards by Wilkinson CP et al. (37), Ophthalmology; 65 citations), alongside studies on the associations of obesity and DR by Man REK et al. (38), Invest Ophthalmol Vis Sci; 63 citations). Influential authors frequently cited together actively build upon these foundational studies to deepen our understanding of the mechanisms underlying DR (**Figure 5D**).

Several key studies, investigating various aspects of DFU, constitute the foundational work in this research area. Among these foundational works, the studies investigating the natural history and recurrence of DFU by Armstrong et al. (39), N Engl J Med) garnered 30 citations. The influential discussion of the global burden of DFU by Boulton et al. (40), Lancet) was cited 21 times. Additionally, clinical care standards outlined by the ADA (2010, Diabetes Care) have garnered 18 citations, while prevention strategies proposed by Singh N et al. (41), JAMA) received 15 citations. Influential co-cited authors (**Figure 5E**) further advanced research on the pathogenesis and management of DFU, building upon this foundational work.

**Figure 6:**
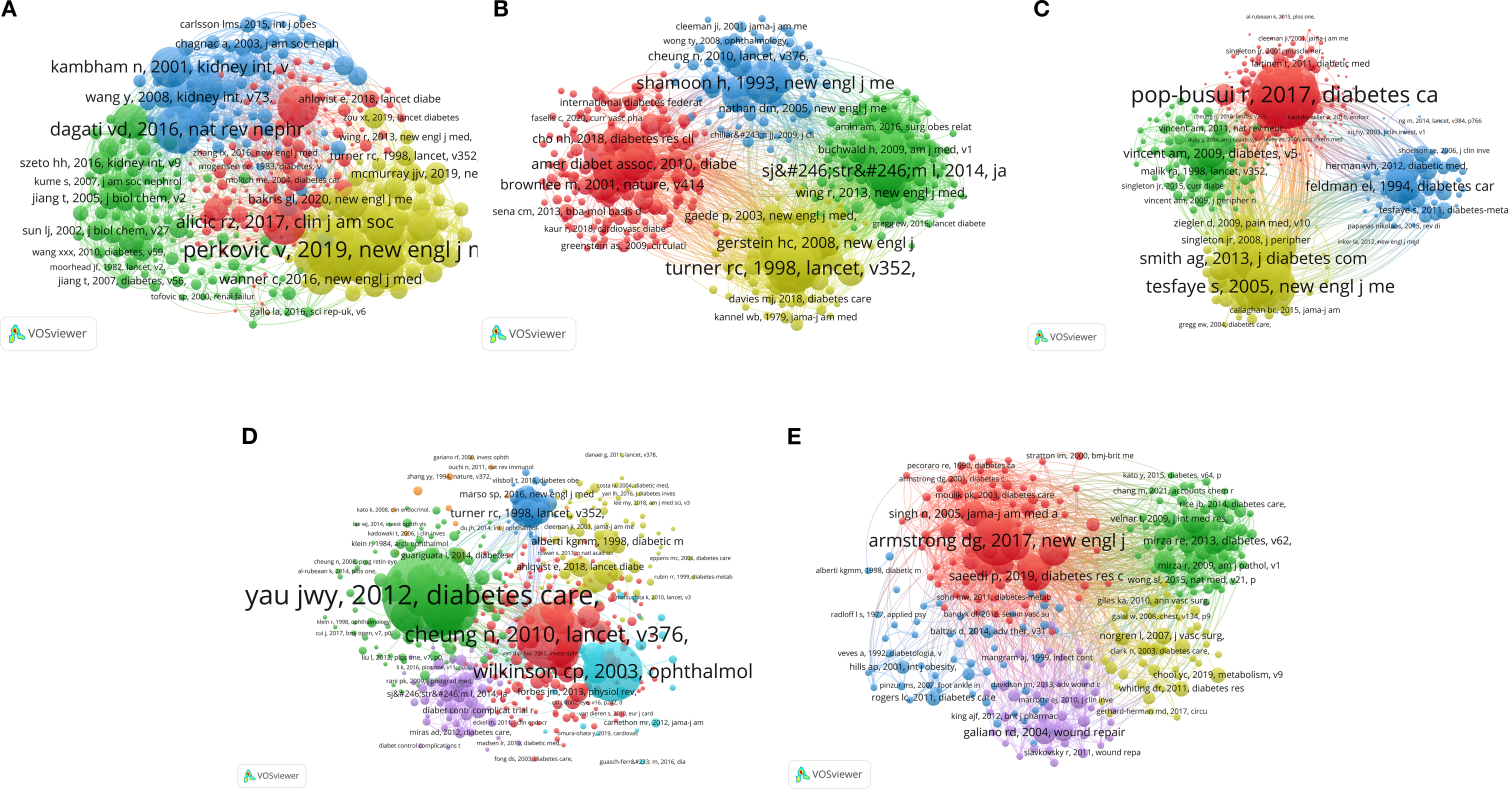
Document co-citation network of studies linking obesity to five major complications of diabetes: **(A)** DKD, **(B)** Diabetic angiopathy, **(C)** DPN, **(D)** DR, and **(E)** DFU.

The composite co-citation network analysis (**Figure 6**) underscored the contributions of leading researchers whose work formed the intellectual backbone of the field. Notable figures were as follows: Perkovic V for his influential DKD trials; Turner RC, and Gerstein HC who contributed significantly to diabetic angiopathy trials and epidemiology; Pop-Busui R and Tesfaye S recognized for their work on DPN guidelines and definitions; Yau JWY, Cheung N, and Klein R who advanced research on the epidemiology of DR; and Armstrong DG and Galiano RD who are known for their contributions to DFU standards and wound healing.

### 3.6 Thematic frameworks and evolving research frontiers

**Figure 7:**
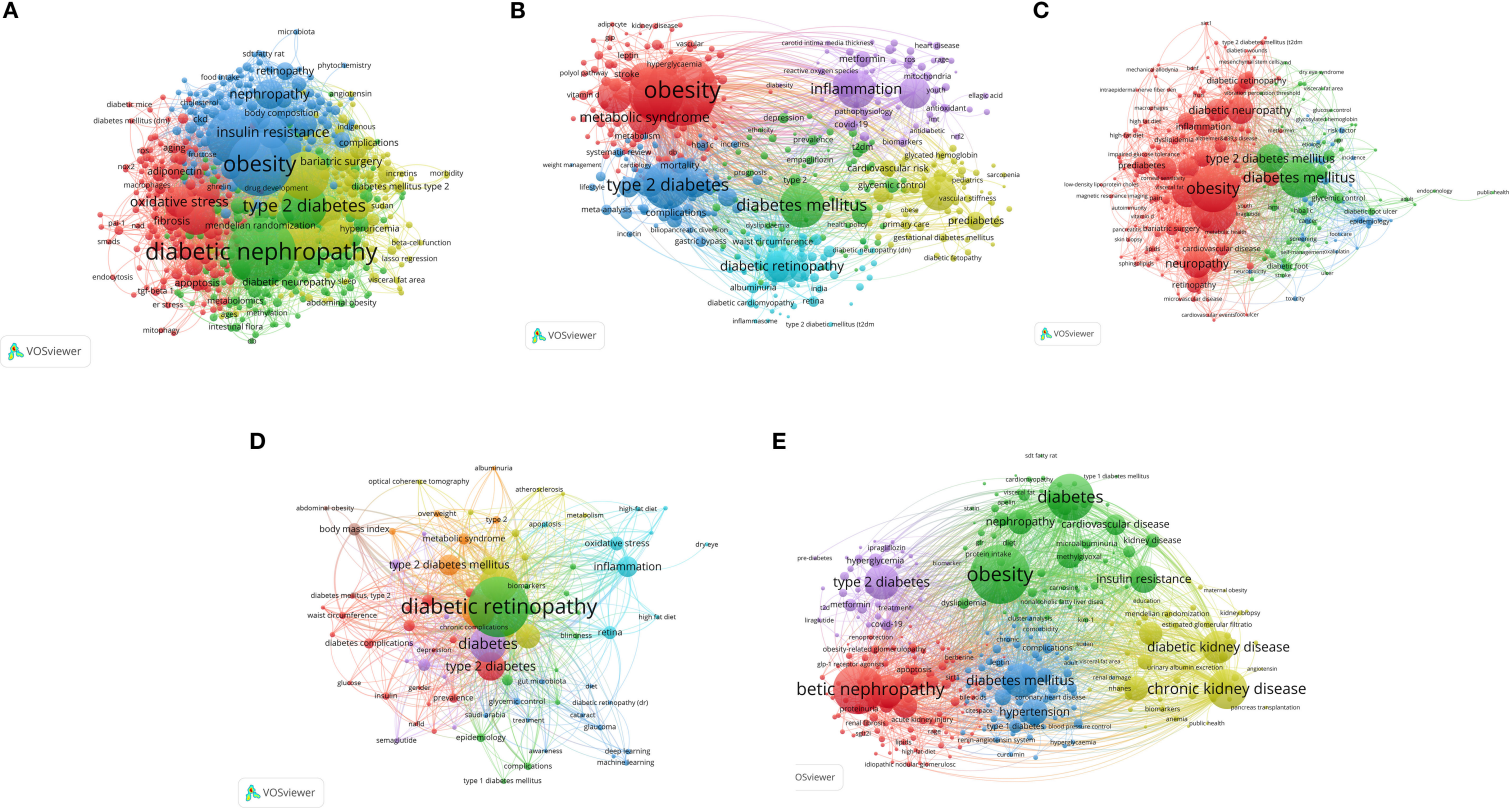
Author keyword co-occurrence network of studies linking obesity to five major complications of diabetes: **(A)** DKD, **(B)** Diabetic angiopathy, **(C)** DPN, **(D)** DR, and **(E)** DFU.

The keyword co-occurrence network analysis of author keywords (**Figure 7**) effectively identified the main conceptual frameworks and research focuses in each subfield of obesity and diabetic complications. This analysis provides valuable insights into the thematic areas that are driving current research, highlighting key terms and their interconnections. By mapping these relationships, researchers can better understand the evolving landscape of study topics and identify emerging trends and gaps in the literature.

Regarding DKD (**Figure 7A**), a central axis linked the “diabetic nephropathy” cluster (green) to the “obesity” and “insulin resistance” clusters (blue). This structure underscores the research focus on the impact of obesity-induced metabolic changes in the progression of diabetic nephropathy, particularly in those with T2DM.

**Figure 7B** provides a systematic research perspective on diabetic angiopathy, highlighting three core interconnected clusters: “obesity” (red, associated with metabolic syndrome), “inflammation” (purple), and “diabetes mellitus” (light blue, linked to complications). The dense connections among these clusters suggest a research paradigm that identifies obesity-driven chronic inflammation as a key pathophysiological link between obesity, diabetes, and vascular complications. This framework underscores the importance of inflammation in the management and prevention of diabetic angiopathy.

**Figure 7C** illustrates a concentrated network structure regarding research on DPN, with most activities focused on a dominant red cluster. The core of this network includes tightly linked nodes, such as “obesity”, “diabetes mellitus”, and “type 2 diabetes”, along with neuropathy-related keywords. This network configuration indicates that the prevailing research paradigm in DPN considers the condition primarily a consequence of the “obesity-diabetes” complex and prioritizes studies on the pathogenesis of nerve damage.

The intellectual structure of DR research, visualized in the keyword network (**Figure 7D**), was built based on the triad of “DR”, “obesity”, and “diabetes”. Extending from this core, a major thematic branch dedicated to clinical and epidemiological profiles was evident, characterized by high-frequency terms, such as “risk factors”, “prevalence”, and “metabolic syndrome”. The other branch, with keywords such as “oxidative stress”, “inflammation”, and “angiogenesis”, provided a deeper insight into the molecular and biological drivers of DR.

In the DFU keyword network (**Figure 7E**), the structural arrangement of thematic clusters revealed a clear pathogenic trajectory originating from obesity. Ulcer, as the primary clinical outcome, formed a central cluster (red); however, its position in the network was heavily affected by two interconnected clusters: one representing ‘obesity’ as the principal risk factor (green), and another detailing the mediating pathological pathways, ‘neuropathy’ and ‘peripheral artery disease’ (blue). This structural arrangement suggests that obesity contributes significantly to the development of DFU by driving these neuropathic and vascular pathologies.

**Figure 8:**
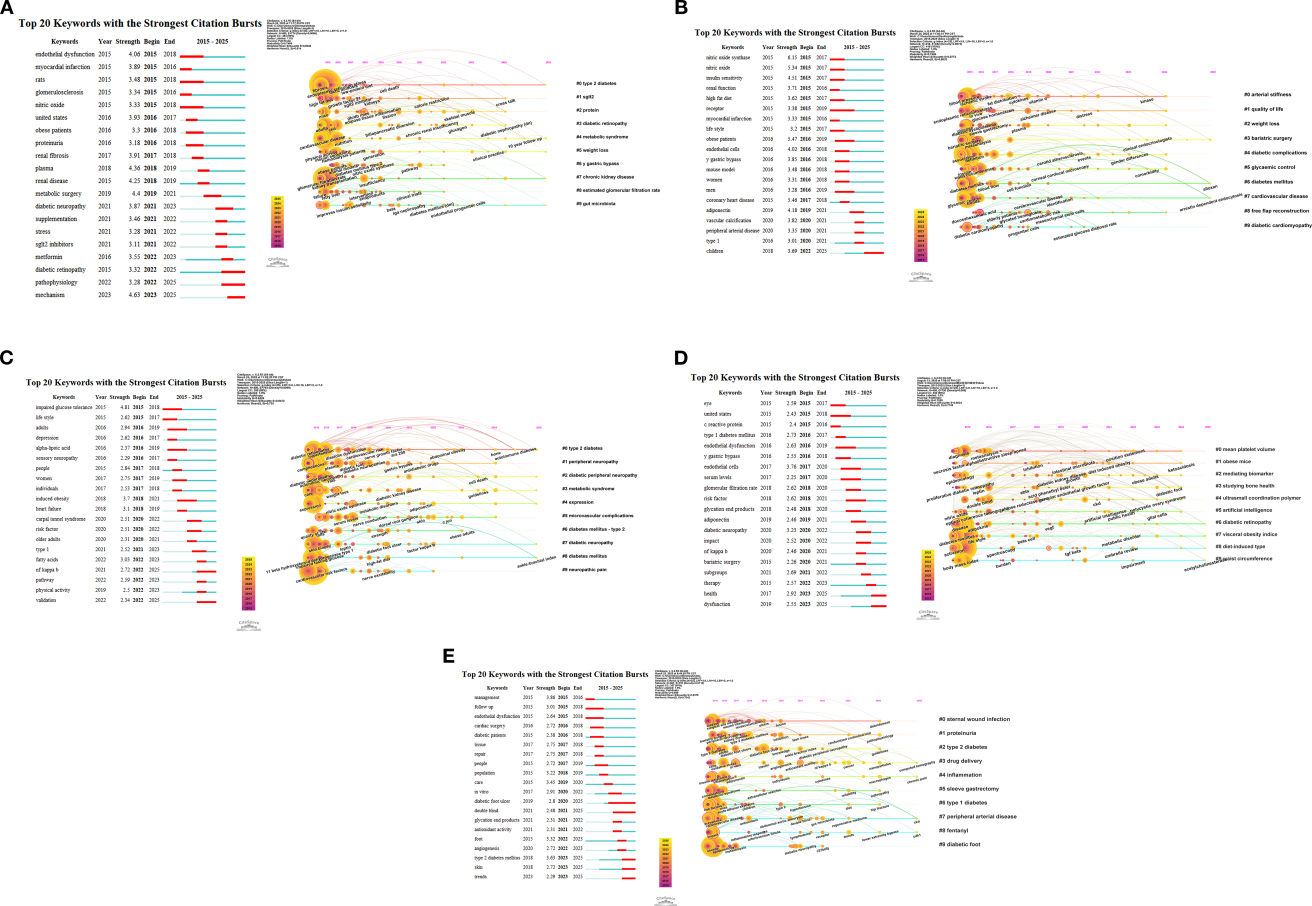
Dynamic trends of the top 20 most frequently cited keywords in research linking obesity to five major diabetic complications: **(A)** DKD, **(B)** Diabetic angiopathy, **(C)** DPN, **(D)** DR, and **(E)** DFU.

Complementing the structural network analysis, a keyword burst detection (**Figure 8**) was employed to provide a dynamic perspective on the evolution of this field from 2015 to 2025, highlighting key shifts in research focus and emerging trends.

The evolving research focus in DKD (**Figure 8A**) began with a strong emphasis on basic pathophysiology and clinical characteristics from 2015 to 2018. This period saw significant bursts in mechanistic terms like ‘endothelial dysfunction’ (4.06) and ‘nitric oxide’ (3.33), alongside key clinical descriptors, such as ‘obese patients’ (3.3) and ‘proteinuria’ (3.18). Subsequently, between 2019 and 2021, the research paradigm shifted markedly from observational characterization toward therapeutic interventions. This transition was exemplified by the powerful burst of the keyword “metabolic surgery” (burst strength: 4.4), suggesting increased interest in high-impact treatment strategies. From 2022 to 2025, research moved toward deeper mechanistic studies, emphasizing “pathophysiology” (3.28) and “mechanism” (strongest recent burst 4.63). Additionally, there was an increasing interest in the links between complications, such as “DR” (3.32), and these terms.

**Figure 8B** outlines the progression of research focus in diabetic angiopathy. From 2015 to 2017, studies focused on basic mechanisms, such as “nitric oxide synthase” (burst strength 6.15). From 2016 to 2019, the focus shifted toward clinical populations, particularly “obese patients” (5.47). Recently, there has been a diversification in research topics, with notable bursts in biomarkers, such as “adiponectin” (4.18, 2019-2021), and specific populations, such as “children” (3.69, 2022-2025). The burst in research concerning “children” was the latest and longest burst, suggesting a significant expansion at the frontier of research on diabetic angiopathy.

DPN - **Figure 8C**: The research evolution in the field of DPN began with an emphasis on early risk factors, such as “impaired glucose tolerance” (burst strength 4.81, 2015-2018) and “lifestyle” (2.62, 2015-2017). This focus then transitioned to mid-term triggers and associations, including “induced obesity” (3.7, 2018-2021) and “heart failure” (3.1, 2018-2019). Recently, attention has shifted toward specific clinical entities like “carpal tunnel syndrome” (2.51, 2020-2022), molecular pathways, such as “NF-kappa B” (2.72, 2022-2025), and methodological rigor exemplified by “validation” (2.34, 2022-2025). The latter two showed sustained bursts in recent years.

DR - **Figure 8D**: The research evolution in DR began with an emphasis on foundational mechanisms, such as “endothelial dysfunction” (burst strength 2.63, 2016-2019) and “glycation-end products” (2.48, 2018-2020). This focus then transitioned to specific biomarkers and interventions, including “adiponectin” (2.46, 2019-2021) and “bariatric surgery” (2.26, 2020-2021). Recently, attention has shifted toward broader patient outcomes and population analysis, with strong and sustained bursts in keywords, like “health” (2.92, 2023-2025), “dysfunction” (2.55, 2023-2025), and “subgroups” (2.69, 2021-2022).

**Figure 8E** illustrates the shifting research focus in studying DFU over time. Initially, the emphasis was on clinical practice aspects, such as “management” (burst strength 3.86, 2015-2016) and “follow-up” (3.01, 2015-2018). In the mid-term, attention shifted to care delivery, highlighted by terms like “care” (3.45, 2019-2020) and the core condition “DFU”, which showed a sustained burst (2.80, 2020-2025). Recently, studies have strongly emphasized the role of the underlying disease “T2DM” (high strength burst 3.63, 2023-2025), tissue involvement in the “skin” (2.72, 2023-2025), and epidemiological trends captured by “trends” (2.29, 2023-2025). These areas represent the current frontiers in DFU research.

### 3.7 Research hotspots and frontiers

**Figure 9:**
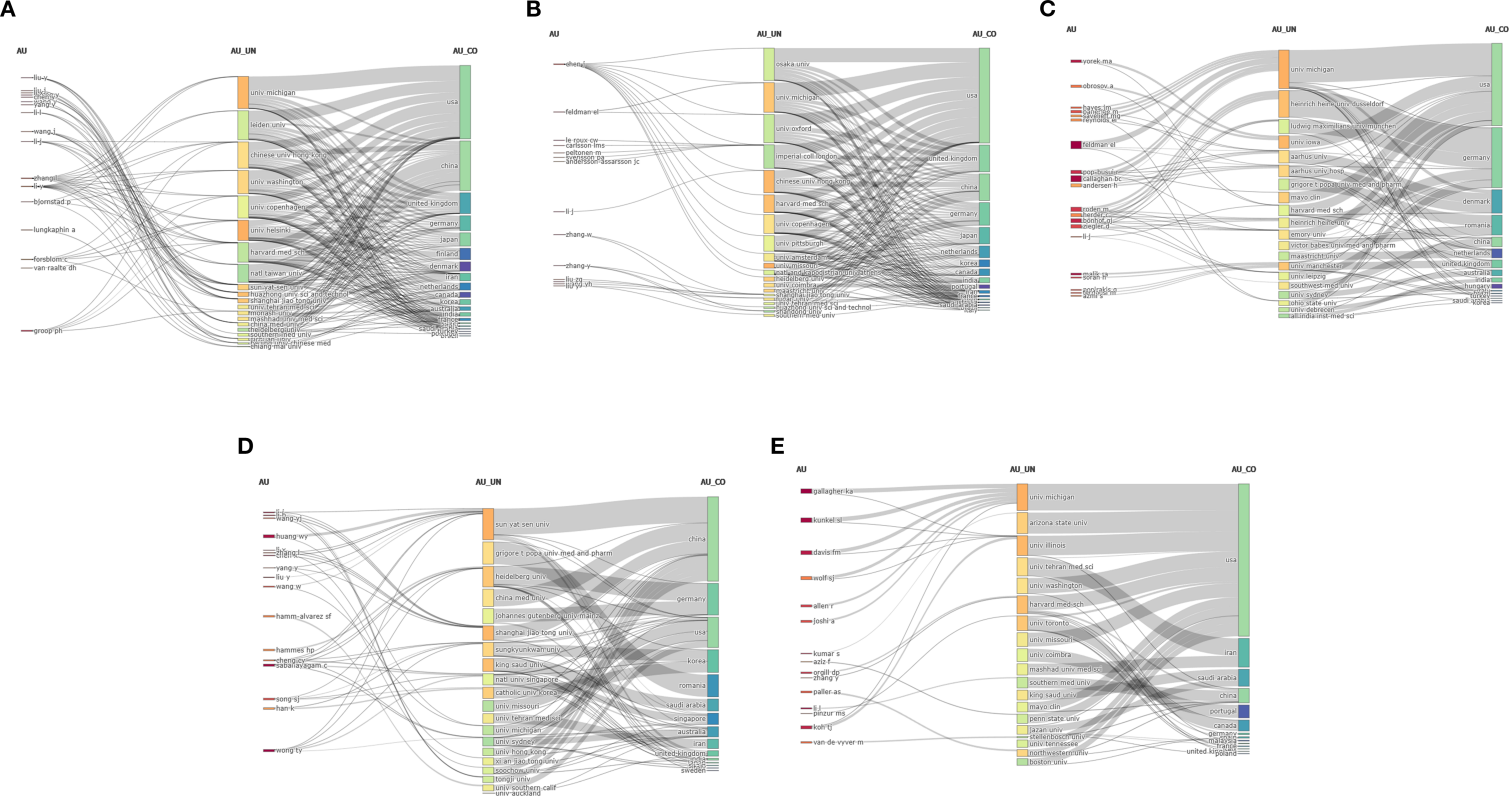
Three-field Sankey diagrams illustrating the collaborative networks of major authors, their institutions, and countries in research linking obesity to five major diabetic complications: **(A)** DKD, **(B)** Diabetic angiopathy, **(C)** DPN, **(D)** DR, and **(E)** DFU.

**Figure 9** illustrates the collaborative networks of major authors, institutions, and countries regarding the five complications. A common feature was the dominant role of the USA, which led in four of the five fields, particularly in diabetic neuropathy (**Figure 9C**) and DFU (**Figure 9D**). In particular, the University of Michigan emerged as a central hub globally. China also demonstrated significant influence, especially in diabetic nephropathy (**Figure 9A**) and possessed a leading position in DR, with Sun Yat-sen University at the forefront of research (**Figure 9E**). European powerhouses, including the United Kingdom and Germany, also played crucial roles, particularly in diabetic angiopathy (**Figure 9B**) and DPN (**Figure 9C**), respectively. The analysis highlighted the contributions of highly prolific authors, such as Feldman EL and Groop PH, whose affiliations with these leading institutions underscore the concentration of academic influence in a few research institutions.

## 4 Discussion

This bibliometric study provided a data-driven narrative of a critical shift in modern diabetology, suggesting the evolution in the role of obesity from a recognized risk factor to a central hub in the pathogenesis of the most severe complications of diabetes. Our analysis of over 5,000 articles did not merely list publications but also quantitatively mapped the intellectual structure, thematic evolution, and collaborative networks of this maturing research paradigm. By systematically dissecting the landscape for each of the five major complications of diabetes, we offer insights into publication trends, major contributors, research hotspots, and future perspectives, offering an overview of the field’s trajectory.

### 4.1 A comparative synthesis of disease-specific research landscapes

Our analysis revealed that each complication of diabetes has cultivated a distinct research ecology, defined by significant variations in its core drivers, structural organization, and intellectual foundations. This diversity was first identified at a macro level. DKD was the most studied complication, reflecting its high clinical burden, while DPN exhibited the most rapid growth trajectory. Structurally, the landscape is shaped by the leadership of the USA and China. China demonstrated particular strength in DKD and DR, whereas the USA excelled in diabetic angiopathy, DPN, and DFU due to the presence of many experts in key institutions like the University of Michigan. Furthermore, these subfields are built upon unique intellectual foundations. For example, research on diabetic angiopathy is heavily shaped by landmark CVOTs. The management of DPN is guided by authoritative clinical position statements, and there are large-scale epidemiological studies regarding DR. DFU is studied by a multidisciplinary cooperation of experts in wound care and clinical diabetology. Despite these profound differences, our analysis showed that over time, the research focus has shifted across all domains. Initially focused on foundational mechanisms, such as ‘endothelial dysfunction’, the field has evolved to prioritize high-impact interventions and studies with greater specificity. Examples of this shift include the surge in ‘metabolic surgery’ for DKD, a sustained focus on ‘children’ in diabetic angiopathy, and deeper mechanistic studies on pathways like ‘NF-kappa B’ in DPN. These evolving frontiers not only underscore the maturation of the field but also necessitate a clear agenda for future research. Key priorities include elucidating the mechanisms mediating the effects of surgical interventions, addressing the emerging crisis of pediatric metabolic disease, and identifying novel therapeutic targets at the molecular level. Despite the distinct research trajectories characterizing each complication, our detailed mapping revealed a clear convergence toward a shared paradigm. This emerging model is defined by an increasing focus on mechanisms, interventions, and precision medicine.

### 4.2 Critical implications for future clinical research

By highlighting the varied maturity across subfields, the bibliometric landscape mapped here provides an essential framework for the strategic reorientation of future clinical research. The maturity of the DKD field, evidenced by its sheer volume, implies that future trials must assess efficacy in general populations. Instead, studies should investigate why significant risk persists in patients already receiving optimal therapy (e.g., SGLT2i/GLP-1RA) and target non-responders. The nascent but rapidly growing DPN domain should advance from small-scale observational studies to rigorously designed, large-scale “Nerve Outcome Trials” (NOTs), mirroring the success and methodological rigor of the CVOTs in the treatment of diabetic angiopathy. Intellectual arbitrage is a powerful strategy emerging from our cross-disease analysis, which applies successful research paradigms from one subfield to another. For instance, it tests the multidisciplinary, integrated care model that underpins DFU research as a formal complex intervention in high-risk DKD or DPN populations. Furthermore, the surge in “metabolic surgery” and “pediatric” research highlights the shift from overall efficacy to personalized medicine, mechanistic assessment, and long-term cost-effectiveness. This necessitates a paradigm shift in trial design, moving away from monolithic, “one-size-fits-all” approaches toward studies designed with *a priori* stratification hypotheses and which embed deep phenotyping, integrating multi-omics and advanced imaging from their inception. Only through such a precision-oriented, mechanism-based, and cross-disciplinary approach can we dismantle the complex nexus of obesity and diabetic complications.

### 4.3 Valuable academic guidance for clinical practice

In addition to guiding future research, our bibliometric map serves as a critical diagnostic tool for contemporary clinical practice, illuminating the chasm between the evolving evidence landscape and established care protocols. Firstly, the distinct yet interconnected research ecosystems challenge the traditional, organ-centric approach to patient management. The demonstrated success of the multidisciplinary paradigm in DFU research provides a powerful academic mandate for its broader application. Clinicians should form “metabolic complication prevention teams” to conduct holistic risk assessments long before a single complication becomes clinically dominant. Secondly, the map empowers clinicians to practice a form of “anticipatory medicine”. The strong and sustained research signals around topics like “metabolic surgery” for DKD or the focus on “children” in diabetic angiopathy are not merely academic curiosities. They are leading indicators of future guideline shifts. Clinicians should proactively incorporate such evidence into patient counseling. For instance, bariatric surgery should not be considered only a weight-loss tool but a potent kidney-protective strategy in appropriate candidates. Finally, our analysis of a field rich with diverse interventional trials underscored the urgent need for a shift in the clinical mindset from a guideline implementer to a “therapeutic strategist”. The era of a linear, one-size-fits-all treatment algorithm has come to an end. Clinicians are now equipped with multiple classes of effective drugs (SGLT2is, GLP-1RAs, etc.), each offering a unique profile of organ protection. Patients’ comprehensive risk profiles should be used to personalize the sequencing and combination of treatments, thereby prioritizing agents that target the patient’s most vulnerable organ system. Finally, this knowledge map encourages a more dynamic and evidence-based approach to treatment, urging practitioners to navigate the complexities of obesity-related complications of diabetes based on the latest research findings.

### 4.4 Strengths, limitations, and core innovation

This study had several key strengths, including its comprehensive scope, which simultaneously mapped the research landscapes of five major complications of diabetes in the context of obesity, enabling a unique cross-disease comparative analysis (Section 4.1). The use of knowledge graph visualization provided a dynamic perspective, moving beyond simple publication counts to reveal intellectual structures and evolutionary trends. However, we must acknowledge several limitations. Although our analysis was based on three major databases (Web of Science, Scopus, and PubMed), our focus was primarily on English-language publications. Therefore, we might have lost non-English studies and those not indexed in the three databases. Most critically, this analysis mapped research activity, not the quality or validity of clinical evidence. Therefore, our findings should be interpreted as a strategic guide to the scientific landscape, serving as a complement to, not a substitute for, evidence synthesis methods like systematic reviews and meta-analyses.

The core innovation of this work, however, transcends the standard application of bibliometric techniques. Although most bibliometric studies provide a descriptive summary of research trends, our study fundamentally reframed the methodology as a diagnostic and prognostic tool for the entire field. The true novelty of this study lies in our analytical framework, which offers three distinct intellectual advantages that distinguish this study from previous works: (1) It establishes a panoramic and comparative framework (Section 4.1), analyzing five interconnected complications in parallel to reveal shared patterns and unique trajectories, thereby enabling the strategic concept of “intellectual arbitrage.” (2) It bridged the chasm between macro-level data and actionable strategy by systematically translating bibliometric signals into prescriptive insights for both future clinical research (Section 4.2) and immediate clinical practice (Section 4.3). (3) It redefined the purpose of a knowledge map, not as a static historical photograph of the field, but as a dynamic navigation system (GPS) for clinicians and researchers. In essence, we not only described the map but also provided a legend and a user manual for navigating the future. This transformative approach, from descriptive mapping to strategic guidance, was the main contribution of our research.

## 5 Conclusion

This study employed a comprehensive bibliometric visualization analysis of the literature from 2015 to 2025 across three major databases to systematically highlight the crucial role of obesity as a central factor affecting research on five main complications of diabetes, including DKD, diabetic angiopathy, DPN, DR, and DFU. The analysis meticulously mapped the growing complexity of the field, research intensity, and significant movement toward multidisciplinary integration. The study measured various research momentum across complications, outlined global collaboration structures, tracked the evolution of the core knowledge base, and represented the research shift from associative description to in-depth mechanistic exploration, targeted intervention, and integrated understanding of complications.

These findings provide a macroscopic and quantitative framework and structural insight into the knowledge landscape of this important domain. The results strongly emphasized the clinical need for integrating proactive, effective, and personalized weight management strategies as a key component in the management of diabetes to prevent, delay, and reduce the burden of the complications of diabetes in the long term. Future studies should provide a deeper understanding of the causal mechanisms, develop and evaluate precision intervention strategies, investigate multi-complication network dynamics, enhance translational scientific efforts, and strengthen international and multidisciplinary collaboration to more effectively address the global health challenge posed by the coexistence of obesity and diabetic complications.

## Data Availability

The original contributions presented in the study are included in the article/**Supplementary Material**. Further inquiries can be directed to the corresponding authors.

## Supplementary material

SUPPLEMENTARY FIGURE S1 Flow diagram of the literature screening and study selection process of studies on five types of diabetic complications and obesity.
