# Supplementary figures and images for: Mapping the research landscape of the interactions between obesity and five major complications of diabetes: a bibliometric analysis using knowledge graph visualization
Source: Front Endocrinol (Lausanne). 2025 Oct 23;16:1626191. doi: 10.3389/fendo.2025.1626191 (PMC12588820; doi:10.3389/fendo.2025.1626191)

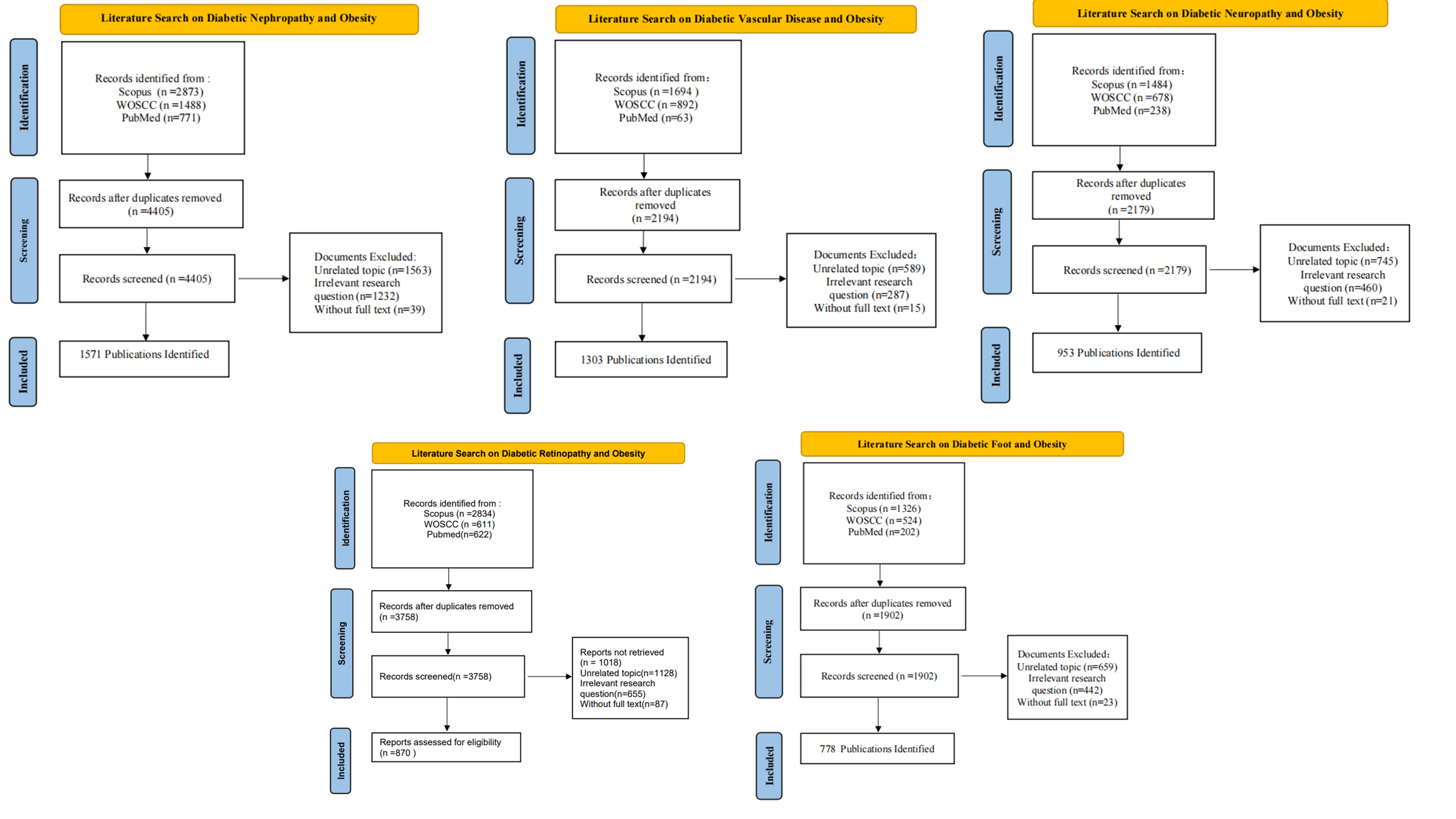

Supplement: SUPPLEMENTARY FIGURE S1 — Flow diagram of the literature screening and study selection process of studies on five types of diabetic complications and obesity. [file Image1.tif]
