# Supplementary material for: Mapping the research landscape of the interactions between obesity and five major complications of diabetes: a bibliometric analysis using knowledge graph visualization
Source: Front Endocrinol (Lausanne). 2025 Oct 23;16:1626191. doi: 10.3389/fendo.2025.1626191 (PMC12588820; doi:10.3389/fendo.2025.1626191)
Supplement: Supplementary file 2 [file DataSheet1.docx]

| Search Strategy | | | |
| --- | --- | --- | --- |
| **Tape** | **WOSCC** | **Scopus** | **Pubmed** |
| Diabetic Kidney Disease (DKD) | ((TS=(obesity)) OR TS=(obese)) OR TS=(overweight )AND(((((((((((TS=(Diabetic Nephropathies)) OR TS=(Nephropathies, Diabetic)) OR TS=(Diabetic Nephropathy)) OR TS=(Nephropathy, Diabetic)) OR TS=(Diabetic Kidney Disease)) OR TS=(Diabetic Glomerulosclerosis)) OR TS=(Intracapillary Glomerulosclerosis)) OR TS=(Kimmelstiel-Wilson Disease)) OR TS=(Kimmelstiel Wilson Disease)) OR TS=(Nodular Glomerulosclerosis)) OR TS=(Kimmelstiel-Wilson Syndrome)) OR TS=(Kimmelstiel Wilson Syndrome) | ( TITLE-ABS-KEY ( obesity ) OR TITLE-ABS-KEY ( obese ) OR TITLE-ABS-KEY ( overweight ) AND TITLE-ABS-KEY ( diabetic AND nephropathies ) OR TITLE-ABS-KEY ( nephropathies, AND diabetic ) OR TITLE-ABS-KEY ( diabetic AND nephropathy ) OR TITLE-ABS-KEY ( nephropathy, AND diabetic ) OR TITLE-ABS-KEY ( diabetic AND kidney AND disease ) OR TITLE-ABS-KEY ( diabetic AND glomerulosclerosis ) OR TITLE-ABS-KEY ( intracapillary AND glomerulosclerosis ) OR TITLE-ABS-KEY ( kimmelstiel-wilson AND disease ) OR TITLE-ABS-KEY ( kimmelstiel AND wilson AND disease ) OR TITLE-ABS-KEY ( nodular AND glomerulosclerosis ) OR TITLE-ABS-KEY ( kimmelstiel-wilson AND syndrome ) OR TITLE-ABS-KEY ( kimmelstiel AND wilson AND syndrome ) ) | (((obesity[Title/Abstract]) OR (obese[Title/Abstract])) OR (overweight[Title/Abstract])) AND ((((((((((((Diabetic Nephropathies[Title/Abstract]) OR (Nephropathies, Diabetic[Title/Abstract])) OR (Diabetic Nephropathy[Title/Abstract])) OR (Nephropathy, Diabetic[Title/Abstract])) OR (Diabetic Kidney Disease[Title/Abstract])) OR (Diabetic Glomerulosclerosis[Title/Abstract])) OR (Intracapillary Glomerulosclerosis[Title/Abstract])) OR (Kimmelstiel-Wilson Disease[Title/Abstract])) OR (Kimmelstiel Wilson Disease[Title/Abstract])) OR (Nodular Glomerulosclerosis[Title/Abstract])) OR (Kimmelstiel-Wilson Syndrome[Title/Abstract])) OR (Kimmelstiel Wilson Syndrome[Title/Abstract])) |
| Diabetic Angiopathy | ((TS=(obesity)) OR TS=(obese)) OR TS=(overweight )AND(((((((((((TS=(Diabetic Angiopathies)) OR TS=(Diabetic Angiopathy)) OR TS=(Angiopathy, Diabetic)) OR TS=(Diabetic Vascular Complications)) OR TS=(Diabetic Vascular Complication)) OR TS=(Vascular Complication, Diabetic)) OR TS=(Vascular Complications, Diabetic)) OR TS=(Diabetic Vascular Disease)) OR TS=(Diabetic Microangiopathy )) OR TS=(Diabetic Macroangiopathy)) OR TS=(Diabetic Microvascular Complications)) OR TS=( Microvascular Complications，Diabetes) | ( TITLE-ABS-KEY ( obesity ) OR TITLE-ABS-KEY ( obese ) OR TITLE-ABS-KEY ( overweight ) AND TITLE-ABS-KEY ( diabetic AND angiopathies ) OR TITLE-ABS-KEY ( diabetic angiopathy ) OR TITLE-ABS-KEY ( angiopathy, AND diabetic ) OR TITLE-ABS-KEY ( diabetic AND vascular AND complications ) OR TITLE-ABS-KEY ( diabetic AND vascular AND complication ) OR TITLE-ABS-KEY ( vascular AND complication, AND diabetic ) OR TITLE-ABS-KEY ( vascular AND complications, AND diabetic ) OR TITLE-ABS-KEY ( diabetic AND vascular AND disease ) OR TITLE-ABS-KEY ( diabetic AND microangiopathy ) OR TITLE-ABS-KEY ( diabetic AND macroangiopathy ) OR TITLE-ABS-KEY ( diabetic AND microvascular AND complications ) OR TITLE-ABS-KEY ( microvascular AND complications ， diabetes ) ) | (((obesity[Title/Abstract]) OR (obese[Title/Abstract])) OR (overweight[Title/Abstract])) AND ((((((((((((Diabetic Angiopathies[Title/Abstract]) OR (Diabetic Angiopathy[Title/Abstract])) OR (Angiopathy, Diabetic[Title/Abstract])) OR (Diabetic Vascular Complications[Title/Abstract])) OR (Diabetic Vascular Complication[Title/Abstract])) OR (Vascular Complication, Diabetic[Title/Abstract])) OR (Vascular Complications, Diabetic[Title/Abstract])) OR (Diabetic Vascular Disease[Title/Abstract])) OR (Diabetic Microangiopathy[Title/Abstract])) OR (Diabetic Macroangiopathy[Title/Abstract])) OR (Diabetic Microvascular Complications[Title/Abstract])) OR (Microvascular Complications,Diabetes[Title/Abstract])) |
| Diabetic Peripheral Neuropathy (DPN) | ((TS=(obesity)) OR TS=(obese)) OR TS=(overweight )AND((((((((((((((((((((TS=(Diabetic Neuropathies)) OR TS=(Diabetic Peripheral Neuropathy)) OR TS=(Neuropathy，Diabetes )) OR TS=(Diabetic Neuropathy )) OR TS=(Neuropathies, Diabetic )) OR TS=(Neuropathy, Diabetic)) OR TS=(Diabetic Amyotrophy)) OR TS=(Amyotrophy, Diabetic)) OR TS=(Diabetic Autonomic Neuropathy)) OR TS=(Autonomic Neuropathies, Diabetic)) OR TS=(Diabetic Neuralgia)) OR TS=(Neuralgias, Diabetic)) OR TS=(Neuralgia, Diabetic)) OR TS=(Neuropathy, Painful Diabetic)) OR TS=(Painful Diabetic Neuropathy)) OR TS=(Diabetic Polyneuropathy)) OR TS=(Diabetic Polyneuropathies)) OR TS=(Polyneuropathies, Diabetic)) OR TS=(Polyneuropathy, Diabetic)) OR TS=(Diabetic Mononeuropathy)) OR TS=(Diabetic Mononeuropathies ) | ( TITLE-ABS-KEY ( obesity ) OR TITLE-ABS-KEY ( obese ) OR TITLE-ABS-KEY ( overweight ) AND TITLE-ABS-KEY ( diabetic AND neuropathies ) OR TITLE-ABS-KEY ( diabetic AND peripheral AND neuropathy ) OR TITLE-ABS-KEY ( neuropathy ， diabetes ) OR TITLE-ABS-KEY ( diabetic AND neuropathy ) OR TITLE-ABS-KEY ( neuropathies, AND diabetic ) OR TITLE-ABS-KEY ( neuropathy, AND diabetic ) OR TITLE-ABS-KEY ( diabetic AND amyotrophy ) OR TITLE-ABS-KEY ( amyotrophy, AND diabetic ) OR TITLE-ABS-KEY ( diabetic AND autonomic AND neuropathy ) OR TITLE-ABS-KEY ( autonomic AND neuropathies, AND diabetic ) OR TITLE-ABS-KEY ( diabetic AND neuralgia ) OR TITLE-ABS-KEY ( neuralgias, AND diabetic ) OR TITLE-ABS-KEY ( neuralgia, AND diabetic ) OR TITLE-ABS-KEY ( neuropathy, AND painful AND diabetic ) OR TITLE-ABS-KEY ( painful AND diabetic AND neuropathy ) OR TITLE-ABS-KEY ( diabetic AND polyneuropathy ) OR TITLE-ABS-KEY ( diabetic AND polyneuropathies ) OR TITLE-ABS-KEY ( polyneuropathies, AND diabetic ) OR TITLE-ABS-KEY ( polyneuropathy, AND diabetic ) OR TITLE-ABS-KEY ( diabetic AND mononeuropathy ) OR TITLE-ABS-KEY ( diabetic AND mononeuropathies ) ) | (((obesity[Title/Abstract]) OR (obese[Title/Abstract])) OR (overweight[Title/Abstract])) AND (((((((((((((((((((((Diabetic Neuropathies[Title/Abstract]) OR (Diabetic Peripheral Neuropathy[Title/Abstract])) OR (Neuropathy,Diabetes[Title/Abstract])) OR (Diabetic Neuropathy[Title/Abstract])) OR (Neuropathies, Diabetic[Title/Abstract])) OR (Neuropathy, Diabetic[Title/Abstract])) OR (Diabetic Amyotrophy[Title/Abstract])) OR (Amyotrophy, Diabetic[Title/Abstract])) OR (Diabetic Autonomic Neuropathy[Title/Abstract])) OR (Autonomic Neuropathies, Diabetic[Title/Abstract])) OR (Diabetic Neuralgia[Title/Abstract])) OR (Neuralgias, Diabetic[Title/Abstract])) OR (Neuralgia, Diabetic[Title/Abstract])) OR (Neuropathy, Painful Diabetic[Title/Abstract])) OR (Painful Diabetic Neuropathy[Title/Abstract])) OR (Diabetic Polyneuropathy[Title/Abstract])) OR (Diabetic Polyneuropathies[Title/Abstract])) OR (Polyneuropathies, Diabetic[Title/Abstract])) OR (Polyneuropathy, Diabetic[Title/Abstract])) OR (Diabetic Mononeuropathy[Title/Abstract])) OR (Diabetic Mononeuropathies[Title/Abstract])) |
| Diabetic Retinopathy (DR) | ((TS=(obesity)) OR TS=(obese)) OR TS=(overweight )AND ((((TS=(Diabetic Retinopathy)) OR TS=(Retinopathy, Diabetic)) OR TS=(Diabetic Macular Edema)) OR TS=(Diabetic Eye Disease) OR TS=(High Glucose,Retin*)) OR TS=(Hyperglycaemic,Retin*) | ( TITLE-ABS-KEY ( obesity ) OR TITLE-ABS-KEY ( obese ) OR TITLE-ABS-KEY ( overweight ) AND TITLE-ABS-KEY ( Diabetic Retinopathy ) OR TITLE-ABS-KEY ( Retinopathy , Diabetic ) OR TITLE-ABS-KEY ( Diabetic Macular Edema ) OR TITLE-ABS-KEY ( Diabetic Eye Disease ) OR TITLE-ABS-KEY ( High Glucose , Retin* ) OR TITLE-ABS-KEY ( Hyperglycaemic , Retin* ) ) | (obesity[Title/Abstract] OR obese[Title/Abstract] OR overweight[Title/Abstract]) AND ("Diabetic Retinopathy"[Title/Abstract] OR "Retinopathy, Diabetic"[Title/Abstract] OR "Diabetic Macular Edema"[Title/Abstract] OR "Diabetic Eye Disease"[Title/Abstract] OR "High Glucose,Retin*"[Title/Abstract] OR "Hyperglycaemic,Retin*"[Title/Abstract]) |
| Diabetic Foot Ulcers (DFU) | ((TS=(overweight)) OR TS=(obesity )) OR TS=(obese )AND((((((((((((TS=(Diabetic Foot)) OR TS=(Diabetic Feet)) OR TS=(Diabetes Foot)) OR TS=(Diabetic Foot Ulcer)) OR TS=(Diabetic Foot Gangrene)) OR TS=(Lower Extremity Peripheral Arterial Disease)) OR TS=(Diabetic Wound)) OR TS=(Diabetic Ulcer)) OR TS=(Foot, Diabetic)) OR TS=(Feet,Diabetic)) OR TS=(Foot Ulcer, Diabetic)) OR TS=(Diabetic Foot Infection)) OR TS=(Diabetic Foot Wounds) | ( TITLE-ABS-KEY ( obesity ) OR TITLE-ABS-KEY ( obese ) OR TITLE-ABS-KEY ( overweight ) AND TITLE-ABS-KEY ( diabetic AND foot ) OR TITLE-ABS-KEY ( diabetic AND feet ) OR TITLE-ABS-KEY ( diabetes AND foot ) OR TITLE-ABS-KEY ( diabetic AND foot AND ulcer ) OR TITLE-ABS-KEY ( diabetic AND foot AND gangrene ) OR TITLE-ABS-KEY ( lower AND extremity AND peripheral AND arterial AND disease ) OR TITLE-ABS-KEY ( diabetic AND wound ) OR TITLE-ABS-KEY ( diabetic AND ulcer ) OR TITLE-ABS-KEY ( foot, AND diabetic ) OR TITLE-ABS-KEY ( feet,diabetic ) OR TITLE-ABS-KEY ( foot AND ulcer, AND diabetic ) OR TITLE-ABS-KEY ( diabetic AND foot AND infection ) OR TITLE-ABS-KEY ( diabetic AND foot AND wounds ) ) | (((obesity[Title/Abstract]) OR (obese[Title/Abstract])) OR (overweight[Title/Abstract])) AND (((((((((((((Diabetic Foot[Title/Abstract]) OR (Diabetic Feet[Title/Abstract])) OR (Diabetes Foot[Title/Abstract])) OR (Diabetic Foot Ulcer[Title/Abstract])) OR (Diabetic Foot Gangrene[Title/Abstract])) OR (Lower Extremity Peripheral Arterial Disease[Title/Abstract])) OR (Diabetic Wound[Title/Abstract])) OR (Diabetic Ulcer[Title/Abstract])) OR (Foot, Diabetic[Title/Abstract])) OR (Feet,Diabetic[Title/Abstract])) OR (Foot Ulcer, Diabetic[Title/Abstract])) OR (Diabetic Foot Infection[Title/Abstract])) OR (Diabetic Foot Wounds[Title/Abstract])) |
