# Supplementary material for: Mapping the research landscape of the interactions between obesity and five major complications of diabetes: a bibliometric analysis using knowledge graph visualization
Source: Front Endocrinol (Lausanne). 2025 Oct 23;16:1626191. doi: 10.3389/fendo.2025.1626191 (PMC12588820; doi:10.3389/fendo.2025.1626191)
Supplement: Supplementary file 3 [file DataSheet2.docx]

| **Supplementary Table S1. Top 10 journals and co-cited journals related to DKD, Diabetic angiopathy, DPN, DR, and DFU.** | | | | | | | | | | | |
| --- | --- | --- | --- | --- | --- | --- | --- | --- | --- | --- | --- |
| **Type** | **Rank** | **Journal** | **Documents** | **Citations** | **IF** | **JCR** | **Rank** | **Co-cited journal** | **Citations** | **IF** | **JCR** |
| **DKD** | **1** | **Plos One** | **39** | **916** | **2.9** | **Q2** | **1** | **Diabetes Care** | **2957** | **14.8** | **Q1** |
|  | **2** | **Frontiers In Endocrinology** | **37** | **458** | **3.9** | **Q1** | **2** | **Kidney Int** | **2468** | **14.8** | **Q1** |
|  | **3** | **International Journal Of Molecular Sciences** | **37** | **1197** | **4.9** | **Q1** | **3** | **J Am Soc Nephrol** | **2198** | **10.3** | **Q1** |
|  | **4** | **Nutrients** | **27** | **1085** | **4.8** | **Q1** | **4** | **Diabetes** | **2114** | **6.2** | **Q1** |
|  | **5** | **Scientific Reports** | **24** | **376** | **3.8** | **Q2** | **5** | **New Engl J Med** | **1896** | **96.2** | **Q1** |
|  | **6** | **American Journal Of Physiology-Renal Physiology** | **20** | **426** | **3.7** | **Q1** | **6** | **Plos One** | **1656** | **2.9** | **Q2** |
|  | **7** | **Frontiers In Pharmacology** | **19** | **224** | **4.4** | **Q1** | **7** | **Diabetologia** | **1403** | **8.4** | **Q1** |
|  | **8** | **Nephrology Dialysis Transplantation** | **19** | **405** | **4.8** | **Q1** | **8** | **Am J Physiol-Renal** | **1202** | **3.7** | **Q1** |
|  | **9** | **Diabetes Research And Clinical Practice** | **18** | **169** | **6.1** | **Q2** | **9** | **Lancet** | **1153** | **98.4** | **Q1** |
|  | **10** | **Journal Of Clinical Medicine** | **18** | **297** | **3.0** | **Q2** | **10** | **Nephrol Dial Transpl** | **1078** | **4.8** | **Q1** |
| **Diabetic angiopathy** | **1** | **Frontiers In Endocrinology** | **29** | **541** | **3.9** | **Q1** | **1** | **Diabetes Care** | **3845** | **14.8** | **Q1** |
|  | **2** | **International Journal Of Molecular Sciences** | **27** | **534** | **4.9** | **Q1** | **2** | **Diabetes** | **2307** | **6.2** | **Q1** |
|  | **3** | **Plos One** | **22** | **683** | **2.9** | **Q2** | **3** | **New Engl J Med** | **1822** | **96.2** | **Q1** |
|  | **4** | **Journal Of Clinical Medicine** | **18** | **275** | **3.0** | **Q2** | **4** | **Diabetologia** | **1633** | **8.4** | **Q1** |
|  | **5** | **Journal Of Diabetes And Its Complications** | **17** | **307** | **3.9** | **Q3** | **5** | **Circulation** | **1477** | **35.5** | **Q1** |
|  | **6** | **Cureus Journal Of Medical Science** | **16** | **68** | **1** | **Q3** | **6** | **Plos One** | **1306** | **2.9** | **Q2** |
|  | **7** | **Nutrients** | **16** | **995** | **4.8** | **Q1** | **7** | **Lancet** | **1203** | **98.4** | **Q1** |
|  | **8** | **Diabetes Research And Clinical Practice** | **15** | **312** | **6.1** | **Q2** | **8** | **Jama-J Am Med Assoc** | **866** | **63.1** | **Q1** |
|  | **9** | **Diabetes & Metabolic Syndrome-Clinical Research & Reviews** | **14** | **128** | **4.3** | **Q1** | **9** | **J Clin Endocr Metab** | **843** | **5.0** | **Q1** |
|  | **10** | **Journal Of Diabetes Research** | **14** | **601** | **3.6** | **Q2** | **10** | **J Clin Invest** | **809** | **13.3** | **Q1** |
| **DPN** | **1** | **Frontiers In Endocrinology** | **26** | **267** | **3.9** | **Q1** | **1** | **Diabetes Care** | **3589** | **14.8** | **Q1** |
|  | **2** | **Cureus Journal Of Medical Science** | **22** | **78** | **1** | **Q3** | **2** | **Diabetes** | **1468** | **6.2** | **Q1** |
|  | **3** | **Journal Of Diabetes Research** | **21** | **562** | **3.6** | **Q2** | **3** | **Diabetologia** | **1146** | **8.4** | **Q1** |
|  | **4** | **Journal Of Diabetes And Its Complications** | **18** | **356** | **3.9** | **Q3** | **4** | **Plos One** | **915** | **2.9** | **Q2** |
|  | **5** | **Plos One** | **18** | **321** | **2.9** | **Q2** | **5** | **New Engl J Med** | **817** | **96.2** | **Q1** |
|  | **6** | **Diabetologia** | **17** | **401** | **8.4** | **Q1** | **6** | **Diabetic Med** | **681** | **3.2** | **Q3** |
|  | **7** | **International Journal Of Molecular Sciences** | **14** | **232** | **4.9** | **Q1** | **7** | **Lancet** | **681** | **98.4** | **Q1** |
|  | **8** | **Scientific Reports** | **13** | **321** | **3.8** | **Q2** | **8** | **Diabetes Res Clin Pr** | **624** | **6.1** | **Q2** |
|  | **9** | **Diabetes Care** | **12** | **887** | **14.8** | **Q1** | **9** | **J Diabetes Complicat** | **524** | **3.9** | **Q3** |
|  | **10** | **Diabetes Research And Clinical Practice** | **11** | **85** | **6.1** | **Q2** | **10** | **J Clin Endocr Metab** | **483** | **5.0** | **Q1** |
| **DR** | **1** | **Plos One** | **25** | **588** | **2.9** | **Q2** | **1** | **Diabetes Care** | **2258** | **14.8** | **Q1** |
|  | **2** | **International Journal Of Molecular Sciences** | **22** | **377** | **4.9** | **Q1** | **2** | **Diabetes** | **1222** | **6.2** | **Q1** |
|  | **3** | **Scientific Reports** | **21** | **304** | **3.8** | **Q2** | **3** | **Plos One** | **1002** | **2.9** | **Q2** |

**Supplementary Table S1. Continued**

| **Type** | **Rank** | **Journal** | **Documents** | **Citations** | **IF** | **JCR** | **Rank** | **Co-cited journal** | **Citations** | **IF** | **JCR** |
| --- | --- | --- | --- | --- | --- | --- | --- | --- | --- | --- | --- |
|  | **4** | **Frontiers In Endocrinology** | **19** | **238** | **3.9** | **Q1** | **4** | **Invest Ophth Vis Sci** | **946** | **5.0** | **Q1** |
|  | **5** | **Diabetes & Metabolic Syndrome-Clinical Research & Reviews** | **14** | **302** | **4.3** | **Q1** | **5** | **Diabetologia** | **911** | **8.4** | **Q1** |
|  | **6** | **Diabetes Research And Clinical Practice** | **14** | **209** | **6.1** | **Q2** | **6** | **Lancet** | **721** | **98.4** | **Q1** |
|  | **7** | **Investigative Ophthalmology & Visual Science** | **14** | **256** | **4.7** | **Q1** | **7** | **New Engl J Med** | **691** | **96.2** | **Q1** |
|  | **8** | **Cureus Journal Of Medical Science** | **12** | **57** | **1** | **Q3** | **8** | **Ophthalmology** | **594** | **13.1** | **Q1** |
|  | **9** | **Journal Of Diabetes Research** | **12** | **194** | **3.6** | **Q2** | **9** | **Diabetes Res Clin Pr** | **576** | **6.1** | **Q2** |
|  | **10** | **BMJ Open Diabetes Research & Care** | **11** | **289** | **4.1** | **Q2** | **10** | **Jama-J Am Med Assoc** | **470** | **63.1** | **Q1** |
| **DFU** | **1** | **Journal Of Foot & Ankle Surgery** | **16** | **81** | **1.3** | **Q4** | **1** | **Diabetes Care** | **1129** | **14.8** | **Q1** |
|  | **2** | **Plos One** | **14** | **442** | **2.9** | **Q2** | **2** | **Plos One** | **534** | **2.9** | **Q2** |
|  | **3** | **Foot & Ankle International** | **10** | **102** | **2.4** | **Q2** | **3** | **Diabetes** | **513** | **6.2** | **Q1** |
|  | **4** | **Diabetes** | **9** | **486** | **6.2** | **Q1** | **4** | **Diabetologia** | **367** | **8.4** | **Q1** |
|  | **5** | **World Journal Of Diabetes** | **9** | **541** | **4.2** | **Q2** | **5** | **New Engl J Med** | **349** | **96.2** | **Q1** |
|  | **6** | **Frontiers In Endocrinology** | **8** | **90** | **3.9** | **Q1** | **6** | **J Vasc Surg** | **342** | **3.9** | **Q1** |
|  | **7** | **Journal Of Diabetes Research** | **8** | **175** | **3.6** | **Q2** | **7** | **Foot Ankle Int** | **320** | **2.4** | **Q2** |
|  | **8** | **Advances In Wound Care** | **7** | **1367** | **5.8** | **Q1** | **8** | **Diabetic Med** | **318** | **3.2** | **Q3** |
|  | **9** | **International Wound Journal** | **6** | **53** | **2.6** | **Q2** | **9** | **Lancet** | **312** | **98.4** | **Q1** |
|  | **10** | **Primary Care Diabetes** | **6** | **205** | **2.6** | **Q1** | **10** | **Wound Repair Regen** | **304** | **3.8** | **Q3** |

| **Supplementary Table S2. Top 10 references related to DKD, Diabetic angiopathy, DPN, DR, and DFU.** | | | | | | | |
| --- | --- | --- | --- | --- | --- | --- | --- |
| **Type** | **Rank** | **Literature** | **Title** | **DOI** | **Source** | **IF/JCR** | **Citations** |
| **DKD** | **1** | **Levey(2009)** | **A new equation to estimate glomerular filtration rate.^[28]^** | **https://doi.org/10.7326/0003-4819-150-9-200905050-00006** | **Ann Intern Med** | **19.6/Q1** | **101** |
|  | **2** | **Alicic(2017)** | **Diabetic Kidney Disease: Challenges, Progress, and Possibilities.^[29]^** | **https://doi.org/10.2215/cjn.11491116** | **Clin J Am Soc Nephro** | **8.5/Q1** | **75** |
|  | **3** | **Dagati(2016)** | **Obesity-related glomerulopathy: clinical and pathologic characteristics and pathogenesis.^[30]^** | **https://doi.org/10.1038/nrneph.2016.75** | **Nat Rev Nephrol** | **72.5/Q1** | **61** |
|  | **4** | **Perkovic(2019)** | **Canagliflozin and Renal Outcomes in Type 2 Diabetes and Nephropathy.^[31]^** | **https://doi.org/10.1056/nejmoa1811744** | **New Engl J Med** | **96.2/Q1** | **61** |
|  | **5** | **Kambham(2001)** | **Obesity-related glomerulopathy: an emerging epidemic.^[32]^** | **https://doi.org/10.1046/j.1523-1755.2001.0590041498.x** | **Kidney Int** | **14.8/Q1** | **49** |
|  | **6** | **Marso(2016)** | **Liraglutide and Cardiovascular Outcomes in Type 2 Diabetes.^[33]^** | **https://doi.org/10.1056/nejmoa1603827** | **New Engl J Med** | **96.2/Q1** | **48** |
|  | **7** | **amer diabet assoc, （2010）** | **Standards of medical care in diabetes--2010.^[34]^** | **https://doi.org/10.2337/dc10-s011** | **Diabetes Care** | **14.8/Q1** | **47** |
|  | **8** | **Neal(2017)** | **Canagliflozin and Cardiovascular and Renal Events in Type 2 Diabetes.^[35]^** | **https://doi.org/10.1056/nejmc1712572** | **New Engl J Med** | **96.2/Q1** | **44** |
|  | **9** | **Sun(2022)** | **IDF Diabetes Atlas: Global, regional and country-level diabetes prevalence estimates for 2021 and projections for 2045.^[36]^** | **https://doi.org/10.1016/j.diabres.2021.109119** | **Diabetes Res Clin Pr** | **6.1/Q2** | **44** |
|  | **10** | **De(2014)** | **Fatty kidney: emerging role of ectopic lipid in obesity-related renal disease.^[37]^** | **https://doi.org/10.1016/s2213-8587(14)70065-8** | **Lancet Diabetes Endo** | **44.0/Q1** | **43** |
| **Diabetic angiopathy** | **1** | **Turner(1998)** | **Intensive blood-glucose control with sulphonylureas or insulin compared with conventional treatment and risk of complications in patients with type 2 diabetes (UKPDS 33).^[38]^** | **10.1016/s0140-6736(98)07019-6** | **Lancet** | **98.4/Q1** | **53** |
|  | **2** | **Marso(2016)** | **Liraglutide and Cardiovascular Outcomes in Type 2 Diabetes. ^[33]^** | **https://doi.org/10.1056/nejmoa1603827** | **New Engl J Med** | **96.2/Q1** | **50** |
|  | **3** | **Stratton(2000)** | **Association of glycaemia with macrovascular and microvascular complications of type 2 diabetes (UKPDS 35): prospective observational study.^[39]^** | **https://doi.org/10.1136/bmj.321.7258.405** | **Bmj-Brit Med J** | **93.7/Q1** | **47** |
|  | **4** | **Sjöström(2014)** | **Association of bariatric surgery with long-term remission of type 2 diabetes and with microvascular and macrovascular complications.^[40]^** | **https://doi.org/10.1001/jama.2014.5988** | **Jama-J Am Med Assoc** | **63.1/Q1** | **46** |
|  | **5** | **Shamoon(1993)** | **The effect of long-term intensified insulin treatment on the development of microvascular complications of diabetes mellitus.^[41]^** | **https://doi.org/10.1056/nejm199307293290502** | **New Engl J Med** | **96.2/Q1** | **45** |
|  | **6** | **Holman(2008)** | **10-year follow-up of intensive glucose control in type 2 diabetes.^[42]^** | **https://doi.org/10.1056/nejmoa0806470** | **New Engl J Med** | **96.2/Q1** | **44** |
|  | **7** | **Gerstein(2008)** | **Effects of Intensive Glucose Lowering in Type 2 Diabetes.^[43]^** | **https://doi.org/10.1056/nejmoa0802743** | **New Engl J Med** | **96.2/Q1** | **36** |
|  | **8** | **Schauer(2017)** | **Bariatric Surgery versus Intensive Medical Therapy for Diabetes - 5-Year Outcomes.^[44]^** | **https://doi.org/10.1056/nejmoa1600869** | **New Engl J Med** | **96.2/Q1** | **36** |
|  | **9** | **Amer Diabet Assoc, (2010)** | **Diagnosis and Classification of Diabetes Mellitus.^[45]^** | **https://doi.org/10.2337/dc11-S062** | **Diabetes Care** | **14.8/Q1** | **34** |
|  | **10** | **Giacco(2010)** | **Oxidative stress and diabetic complications.^[46]^** | **https://doi.org/10.1161/circresaha.110.223545** | **Circ Res** | **16.5/Q1** | **34** |

**Supplementary Table S2. Continued**

| **Type** | **Rank** | **Literature** | **Title** | **DOI** | **Source** | **IF/JCR** | **Citations** |
| --- | --- | --- | --- | --- | --- | --- | --- |
| **DPN** | **1** | **Pop-Busui(2017)** | **Diabetic Neuropathy: A Position Statement by the American Diabetes Association.^[47]^** | **https://doi.org/10.2337/dc16-2042** | **Diabetes Care** | **14.8/Q1** | **103** |
|  | **2** | **Tesfaye(2010)** | **Diabetic neuropathies: update on definitions, diagnostic criteria, estimation of severity, and treatments.^[48]^** | **https://doi.org/10.2337/dc10-1303** | **Diabetes Care** | **14.8/Q1** | **78** |
|  | **3** | **Tesfaye(2005)** | **Vascular risk factors and diabetic neuropathy.^[49]^** | **https://doi.org/10.1056/nejmoa032782** | **N Engl J Med** | **96.2/Q1** | **63** |
|  | **4** | **Smith(2013)** | **Obesity and hyperlipidemia are risk factors for early diabetic neuropathy.^[50]^** | **https://doi.org/10.1016/j.jdiacomp.2013.04.003** | **J Diabetes Complications** | **2.9/Q3** | **57** |
|  | **5** | **Callaghan(2018)** | **Diabetes and obesity are the main metabolic drivers of peripheral neuropathy.^[51]^** | **https://doi.org/10.1002/acn3.531** | **Ann Clin Transl Neurol** | **4.4/Q1** | **55** |
|  | **6** | **Callaghan(2016)** | **Association Between Metabolic Syndrome Components and Polyneuropathy in an Obese Population.^[52]^** | **https://doi.org/10.1001/jamaneurol.2016.3745** | **Jama Neurol** | **20.4/Q1** | **54** |
|  | **7** | **Callaghan(2012)** | **Enhanced glucose control for preventing and treating diabetic neuropathy.^[53]^** | **https://doi.org/10.1002/14651858.cd007543.pub2** | **Cochrane Database Syst Rev** | **8.8/Q1** | **52** |
|  | **8** | **Callaghan(2016)** | **Metabolic Syndrome Components Are Associated With Symptomatic Polyneuropathy Independent of Glycemic Status.^[54]^** | **https://doi.org/10.2337/dc16-0081** | **Diabetes Care** | **14.8/Q1** | **52** |
|  | **9** | **Andersen(2018)** | **Risk Factors for Incident Diabetic Polyneuropathy in a Cohort With Screen-Detected Type 2 Diabetes Followed for 13 Years: ADDITION-Denmark.^[55]^** | **https://doi.org/10.2337/dc17-2062** | **Diabetes Care** | **14.8/Q1** | **42** |
|  | **10** | **Feldman(2019)** | **Diabetic neuropathy.^[56]^** | **https://doi.org/10.1038/s41572-019-0092-1** | **Nat Rev Dis Primers** | **76.9/Q1** | **42** |
| **DR** | **1** | **Yau(2012)** | **Global prevalence and major risk factors of diabetic retinopathy.^[57]^** | **https://doi.org/10.2337/dc11-1909** | **Diabetes Care** | **14.8/Q1** | **97** |
|  | **2** | **Cheung(2010)** | **Diabetic retinopathy.^[58]^** | **https://doi.org/10.1016/s0140-6736(09)62124-3** | **Lancet** | **98.4/Q1** | **73** |
|  | **3** | **Wilkinson(2003)** | **Proposed international clinical diabetic retinopathy and diabetic macular edema disease severity scales.^[59]^** | **https://doi.org/10.1016/s0161-6420(03)00475-5** | **Ophthalmology** | **13.1/Q1** | **65** |
|  | **4** | **Man(2016)** | **Differential Association of Generalized and Abdominal Obesity With Diabetic Retinopathy in Asian Patients With Type 2 Diabetes.^[60]^** | **https://doi.org/10.1001/jamaophthalmol.2015.5103** | **Jama Ophthalmol** | **7.8/Q1** | **63** |
|  | **5** | **Van(2002)** | **Blood pressure, lipids, and obesity are associated with retinopathy - The Hoorn study.^[61]^** | **https://doi.org/10.2337/diacare.25.8.1320** | **Diabetes Care** | **14.8/Q1** | **44** |
|  | **6** | **Dirani(2011)** | **Are Obesity and Anthropometry Risk Factors for Diabetic Retinopathy?: The Diabetes Management Project.^[62]^** | **https://doi.org/10.1167/iovs.11-7208** | **Invest Ophth Vis Sci** | **5.0/Q1** | **42** |
|  | **7** | **Raman(2010)** | **Association of obesity with diabetic retinopathy: Sankara Nethralaya Diabetic Retinopathy Epidemiology and Molecular Genetics Study (SN-DREAMS Report no. 8).^[63]^** | **https://doi.org/10.1007/s00592-009-0113-8** | **Acta Diabetol** | **3.1/Q2** | **36** |
|  | **8** | **Lim(2010)** | **C-reactive protein, body mass index, and diabetic retinopathy.^[64]^** | **https://doi.org/10.1167/iovs.09-4939** | **Invest Ophth Vis Sci** | **5.0/Q1** | **33** |
|  | **9** | **Zhang(2010)** | **Prevalence of diabetic retinopathy in the United States, 2005-2008.^[65]^** | **https://doi.org/10.1001/jama.2010.1111** | **Jama-J Am Med Assoc** | **63.1/Q1** | **33** |
|  | **10** | **Wong(2006)** | **Diabetic retinopathy in a multi-ethnic cohort in the United States.^[66]^** | **https://doi.org/10.1016/j.ajo.2005.08.063** | **Am J Ophthalmol** | **4.1/Q1** | **32** |
| **DFU** | **1** | **Armstrong(2017)** | **Diabetic Foot Ulcers and Their Recurrence.^[67]^** | **https://doi.org/10.1056/nejmra1615439** | **New Engl J Med** | **96.2/Q1** | **30** |
|  | **2** | **Boulton(2005)** | **The global burden of diabetic foot disease.^[68]^** | **https://doi.org/10.1016/s0140-6736(05)67698-2** | **Lancet** | **98.4/Q1** | **21** |
|  | **3** | **Guo(2010)** | **Factors affecting wound healing.^[69]^** | **https://doi.org/10.1177/0022034509359125** | **J Dent Res** | **5.7/Q1** | **21** |
|  | **4** | **Amer(2010)** | **Standards of medical care in diabetes--2010.^[34]^** | **https://doi.org/10.2337/dc10-s011** | **Diabetes Care** | **14.8/Q1** | **18** |

**Supplementary Table S2. Continued**

| **Type** | **Rank** | **Literature** | **Title** | **DOI** | **Source** | **IF/JCR** | **Citations** |
| --- | --- | --- | --- | --- | --- | --- | --- |
|  | **5** | **Saeedi(2019)** | **Global and regional diabetes prevalence estimates for 2019 and projections for 2030 and 2045: Results from the International Diabetes Federation Diabetes Atlas, 9th edition.^[70]^** | **https://doi.org/10.1016/j.diabres.2019.107843** | **Diabetes Res Clin Pract** | **6.1/Q2** | **17** |
|  | **6** | **Sen(2009)** | **Human skin wounds: a major and snowballing threat to public health and the economy.^[71]^** | **https://doi.org/10.1111/j.1524-475x.2009.00543.x** | **Wound Repair Regen** | **3.8/Q3** | **17** |
|  | **7** | **Zhang(2017)** | **Global epidemiology of diabetic foot ulceration: a systematic review and meta-analysis.^[72]^** | **https://doi.org/10.1080/07853890.2016.1231932** | **Ann Med** | **4.9/Q2** | **17** |
|  | **8** | **Cho(2018)** | **IDF Diabetes Atlas: Global estimates of diabetes prevalence for 2017 and projections for 2045.^[73]^** | **https://doi.org/10.1016/j.diabres.2018.02.023** | **Diabetes Res Clin Pract** | **6.1/Q2** | **16** |
|  | **9** | **Singh(2005)** | **Preventing foot ulcers in patients with diabetes.^[74]^** | **https://doi.org/10.1001/jama.293.2.217** | **Jama-J Am Med Assoc** | **63.1/Q1** | **15** |
|  | **10** | **Barrientos(2008)** | **Growth factors and cytokines in wound healing.^[75]^** | **https://doi.org/10.1111/j.1524-475x.2008.00410.x** | **Wound Repair Regen** | **3.8/Q3** | **14** |
